# Occurrence of *mcr-1* and *mcr-2* colistin resistance genes in porcine *Escherichia coli* isolates (2010–2020) and genomic characterization of *mcr-2*-positive *E. coli*

**DOI:** 10.3389/fmicb.2022.1076315

**Published:** 2022-12-09

**Authors:** Christa Ewers, Lisa Göpel, Ellen Prenger-Berninghoff, Torsten Semmler, Katharina Kerner, Rolf Bauerfeind

**Affiliations:** ^1^Faculty of Veterinary Medicine, Institute of Hygiene and Infectious Diseases of Animals, Justus Liebig University Giessen, Giessen, Germany; ^2^NG1 Microbial Genomics, Robert Koch Institute, Berlin, Germany

**Keywords:** mobile colistin resistance, *mcr-2*, *Escherichia coli*, swine, pathotype, plasmid, IncP, IncX4

## Abstract

**Introduction:**

The global emergence of plasmid-mediated colistin resistance is threatening the efficacy of colistin as one of the last treatment options against multi-drug resistant Gram-negative bacteria. To date, ten *mcr*-genes (*mcr-1* to *mcr-10*) were reported. While *mcr-1* has disseminated globally, the occurrence of *mcr-2* was reported scarcely.

**Methods and results:**

We determined the occurrence of *mcr-1* and *mcr-2* genes among *Escherichia coli* isolates from swine and performed detailed genomic characterization of *mcr-2*-positive strains. In the years 2010-2017, 7,614 porcine *E. coli* isolates were obtained from fecal swine samples in Europe and isolates carrying at least one of the virulence associated genes predicting Shiga toxin producing *E. coli* (STEC), enterotoxigenic *E. coli* (ETEC) or enteropathogenic *E. coli* (EPEC) were stored. 793 (10.4%) of these isolates carried the *mcr-1* gene. Of 1,477 additional *E. coli* isolates obtained from sheep blood agar containing 4 mg/L colistin between 2018 and 2020, 36 (2.4%) isolates were *mcr-1*-positive. In contrast to *mcr-1*, the *mcr-2* gene occurred at a very low frequency (0.13%) among the overall 9,091 isolates. Most *mcr-2*-positive isolates originated from Belgium (*n* = 9), one from Spain and two from Germany. They were obtained from six different farms and revealed multilocus sequence types ST10, ST29, ST93, ST100, ST3057 and ST5786. While the originally described *mcr-2.1* was predominant, we also detected a new *mcr-2* variant in two isolates from Belgium, which was termed *mcr-2.8*. MCR-2 isolates were mostly classified as ETEC or ETEC-like, while one isolate from Spain represented an atypical enteropathogenic *E. coli* (aEPEC; *eae*+). The ST29-aEPEC isolate carried *mcr-2* on the chromosome. Another eight isolates carried their *mcr-2* gene on IncX4 plasmids that resembled the pKP37-BE MCR-2 plasmid originally described in Belgium in 2015. Three ST100 *E. coli* isolates from a single farm in Belgium carried the *mcr-2.1* gene on a 47-kb self-transmissible IncP type plasmid of a new IncP-1 clade.

**Discussion:**

This is the first report of *mcr-2* genes in *E. coli* isolates from Germany. The detection of a new *mcr-2* allele and a novel plasmid backbone suggests the presence of so far undetected *mcr-2* variants and mobilizable vehicles.

## Introduction

Antimicrobial resistance against colistin has emerged worldwide and poses a serious challenge to the treatment of diseases caused by multidrug resistant Gram-negative bacteria. The value of colistin as a last resort antimicrobial is compromised by the occurrence of mobile colistin resistance (*mcr*) genes (Schwarz and Johnson, 2016). After the first description of plasmid-encoded *mcr-1* from *Escherichia coli* and *Klebsiella pneumoniae* isolated from patients, food, and animals in China in 2015 (Liu et al., 2016), nine additional *mcr* genes (*mcr-2* to *mcr-10*) and their products were described in different Gram-negative bacterial species (Xavier et al., 2016; AbuOun et al., 2017; Borowiak et al., 2017; Carattoli et al., 2017; Yin et al., 2017; Wang et al., 2018; Yang et al., 2018; Carroll et al., 2019; Wang et al., 2020a).

Since the first report of plasmid-borne *mcr-1* gene, MCR-1-producing *Enterobacterales*, mostly *Escherichia coli*, have been described to occur with different frequencies in livestock, companion animals, wildlife, food and humans across the globe (Ewers et al., 2016; Falgenhauer et al., 2016; Irrgang et al., 2016; Schwarz and Johnson, 2016; Zhang et al., 2016; Unger et al., 2017; Nang et al., 2019). Much less is known about the occurrence of the other *mcr* genes, particularly about *mcr-2* in Europe. MCR-2 is a member of the MCR-family of bacterial phosphoethanolamine transferases and shares 80.6% amino acid identity with MCR-1. The *mcr-2* gene was discovered in some *E. coli* isolates from diarrheic pigs and calves in Belgium (Xavier et al., 2016). It was located on an IncX4 incompatibility-type plasmid (pKP37-BE) of 35,104 bp in size, and it was harbored by mobile insertion element IS*Ec69* which belongs to the IS*1595* insertion sequence family (Xavier et al., 2016). This plasmid was identified in two porcine ST10 *E. coli* isolates and in one bovine ST167 isolate in the original publication.

A similar plasmid has, to the best of our knowledge, only been reported in two further studies. Among 105 colistin-resistant *Salmonella* isolates collected from 2012 to 2015 in the national surveillance program in Belgium, Garcia-Graells and co-authors identified a *mcr-2*-carrying plasmid in a *Salmonella* Derby strain isolated from a pork carcass in 2012 that was almost identical to pKP37-BE (Garcia-Graells et al., 2018). Timmermans et al. (2021) detected *mcr-2* on a pKP37-like IncX4 plasmid in a colistin-resistant *mcr-2*-positive *E. coli* strain from a fattening pig, also from Belgium, in 2016 (Timmermans et al., 2021). Apart from the findings in Belgium, the *mcr-2* gene was only scarcely detected in other European countries, such as Spain, Great Britain and Italy (Dobrzanska et al., 2020; Miguela-Villoldo et al., 2020). In contrast, various studies from non-European countries, predominantly from Asia, confirmed the occurrence of *mcr-2* in Gram-negative bacteria not only from animal (Dutta et al., 2018; Zhang et al., 2018b; Ahmed et al., 2019; Rhouma et al., 2019; Zhang et al., 2019; Islam et al., 2020; Javed et al., 2020; Cilia et al., 2021; Ketkhao et al., 2021) but also from human sources (Zhang et al., 2018a; Ahmed et al., 2019; Mitra et al., 2020; Ara et al., 2021; Ejaz et al., 2021; Imtiaz et al., 2021; Stosic et al., 2021).

Soon after the discovery of *mcr-2*, Poirel et al. (2017) identified a novel allele of *mcr-2* on the chromosome of a *Moraxella pluranimalium* strain isolated from a pig in Spain. Based on molecular data, they proposed that this gene, termed *mcr-2.2*, was likely the progenitor of the *mcr-2* gene identified in Belgium, thereafter designated as *mcr-2.1*. As of 2nd July 2021, seven *mcr-2* alleles (*mcr-2.1* to *mcr-2.7*) are available in the Bacterial Antimicrobial Resistance Reference Gene Database.^1^

In veterinary medicine, colistin has been widely used for the control of neonatal and post-weaning diarrhea in pigs, mainly caused by enterotoxigenic *Escherichia coli* (ETEC) (Luppi et al., 2016; Rhouma et al., 2017). In our microbiological diagnostic laboratory, we receive a large number of samples from pigs for *E. coli* pathotyping and have created an extensive collection of putative pathogenic *E. coli* from pigs in Germany and other European countries in the last decade. In this study, we aimed to examine these *E. coli* isolates for the presence of *mcr-1* and *mcr-2* genes and to perform a detailed characterization of *mcr-2*-positive strains with respect to clonal lineage, *mcr-2* gene variant and location, plasmid types and pathotype of the bacterial host.

## Materials and methods

### Bacterial isolates

*Escherichia coli* isolates (*n* = 9,091) had been obtained from feces or mucosal swabs (rectum or small intestine) collected from pigs during routine microbiological diagnostics at our institute from 2010 through 2020. Samples were collected mainly in Germany (*n* = 7,155) and in 17 other European countries including the Netherlands (*n* = 888), Poland (*n* = 349), Denmark (*n* = 145), Switzerland (*n* = 141), Belgium (*n* = 131), Austria (*n* = 87), Hungary (*n* = 56), Italy (*n* = 42), Spain (*n* = 28), Portugal (*n* = 28), and seven other countries (*n* = 41) (Table 1). The majority of samples had been collected from piglets suffering from post weaning diarrhea or edema disease.

**TABLE 1:** Frequency of *mcr-1* and *mcr-2* in *E. coli* isolates collected from pigs in Germany and other European countries (2010 – 2020).

Country	2010–2017					2018–2020					
	*E. coli* isolates	*mcr-1*		*mcr-2*		*E. coli* isolates	Col non-S*	*mcr-1*			*mcr-2*
	n	n	%	n	%	n	n	n	% Col-non-S	% all	n
Germany	6,158	707	11.5	2	0.03	997	64	36	56.3	3.6	0
The Netherlands	757	3	0.4	0	0	131	0	0	0	0	0
Denmark	140	1	0.7	0	0	5	0	0	0	0	0
Switzerland	129	0	0	0	0	12	0	0	0	0	0
Belgium	113	11	9.7	9	8.0	18	0	0	0	0	0
Poland	102	9	8.8	0	0	247	0	0	0	0	0
Austria	73	0	0	0	0	14	0	0	0	0	0
Spain	28	16	57.1	1	3.6	0	0	0	0	0	0
Portugal	28	17	60.7	0	0	0	0	0	0	0	0
Italy	42	25	59.5	0	0	0	0	0	0	0	0
Hungary	12	4	33.3	0	0	44	0	0	0	0	0
Other countries**	32	0	0	0	0	9	0	0	0	0	0
Total	7,614	793	10.4	12	0.2	1,477	64	36	56.3	2.4	0

*Col non-S: *E. coli* isolates show growth on sheep blood agar containing 4 mg/L colistin.

**United Kingdom (13), Luxembourg (5), Norway (5), Ireland (4), Greek (3), Romania (2), and Slovenia (9).

Porcine *E. coli* isolates were screened for *mcr-1* and *mcr-2* following two different approaches. In a first approach, we included 7,614 *E. coli* isolates that were archived in our institute’s strain collection from 2010 to 2017. Only those isolates had been stored that proved positive by a modified multiplex polymerase chain reaction (PCR) for the gene of at least one of the following virulence factors: adhesive fimbriae F4, F5, F6, F18 and F41; intimin; heat-labile/stable *E. coli* enterotoxins LT-I, ST-I, ST-II; Shiga toxins of type Stx2 (Casey and Bosworth, 2009). These factors are related with enterotoxigenic *E. coli* (ETEC), enteropathogenic *E. coli* (EPEC), and shiga toxin producing *E. coli* (STEC). Cultivation of samples was performed on non-selective media, namely sheep blood agar without antibiotics. Basically, one isolate per pig was stored. The maximum number of samples per farm was limited to 6 pigs per submission. In case isolates from the same pig revealed different virulence gene profiles, one representative isolate of each profile was stored.

In a second approach, we screened 1,477 *E. coli* isolates. The isolates were obtained from fecal and mucosal samples from swine that were sent for routine PCR-based pathotyping to our diagnostic laboratory from May 2018 to December 2020. They were predominantly from Germany (*n* = 997) and from eight other European countries (Table 1). The isolates were streaked on sheep blood agar containing 4 mg/L colistin and were incubated at 37°C overnight. All isolates that showed growth, irrespective of the presence or absence of virulence-associated genes (VAGs), were stored in a glycerin stock at –80°C until further use.

### Polymerase chain reaction screening for *mcr-1* and *mcr-2* genes and clonal grouping of *mcr-2*-positive isolates by pulsed-field gel electrophoresis

*Escherichia coli* isolates collected from 2010 to 2017 were separately cultured overnight in lysogeny broth. Broth cultures of 10 *E. coli* isolates at a time were pooled and screened for *mcr-1* and *mcr-2* using recently published primers and protocols (Liu et al., 2016; Xavier et al., 2016). In case pooled material gave a positive PCR result, single isolates were separately tested by the same PCRs. If pooled material was tested negative, all respective isolates were regarded as negative. *E. coli* isolated from colistin selective media were individually tested for *mcr-1* and *mcr-2* as described before. Prior to whole genome sequencing *mcr-2*-positive *E. coli* isolates were submitted to macrorestriction analysis (*Xba*I) to determine their clonal identity according to a previously published protocol (Ewers et al., 2014). *Xba*I-generated PFGE profiles were compared using BioNumerics software (Version 6.6, Applied Maths, Belgium) and cluster analysis of Dice similarity indices based on UPGMA.

### Whole genome sequencing

DNA for whole genome sequencing was extracted from *E. coli* isolates using the DNA Blood & Tissue Kit according to the manufacturer‘s instruction (Qiagen, Hilden, Germany), followed by library preparation, using Nextera XT library (Illumina, San Diego, USA). DNA was sequenced using Illumina HiSeq 1500 with multiplexing of 70 samples per flow cell using 250 bp paired end reads with a coverage >90 x. Raw reads were adapter-trimmed by Flexbar v.3.0.3 (Resource Identification Portal RRID:SCR_013001), corrected using BayesHammer and assembled *de novo* using SPAdes v3.12.1 (RRID:SCR_000131). Assembled draft genomes were annotated using Prodigal (Prodigal, RRID: SCR_011936).

### Antimicrobial susceptibility testing and resistance gene screening

Minimum inhibitory concentrations (MICs) of colistin were determined by the broth microdilution method according to EUCAST guidelines.^2^ The isolates were further evaluated against 17 other antimicrobial agents by using the VITEK2 compact system (AST-GN38, AST-N248; bioMérieux, Nürtingen, Germany). Results were interpreted according to EUCAST break point tables (EUCAST, 2020) for tigecycline and polymyxin B and according to CLSI guidelines (CLSI, 2018) for the remaining antimicrobial agents.

The web-based tool ResFinder 4.1,^3^ hosted at the Center for Genomic Epidemiology (CGE), was used to identify resistance genes and chromosomal mutations related to β-lactam (*ampC* promoter mutation), fluoroquinolone (mutations in *gyrA*, *gyrB*, *parA*, and *parC*), and colistin resistance (*pmrAB*) based on WGS data (Zankari et al., 2012). Resistance gene screening was carried out by BLASTn (90% identity and 90% query coverage) analysis against homologous genes present in the Comprehensive Antibiotic Resistance Database.^4^

### Genomic location of *mcr-2* and transconjugation assays

The genomic location of the *mcr-2* gene was verified by S1 nuclease digestion of genomic DNA followed by electrophoretic separation and Southern hybridization. A digoxigenin-labeled DNA probe (“DIG luminescent detection Kit”, Boehringer Mannheim GmbH, Mannheim) targeting a 567-bp PCR fragment specific for the *mcr-2* gene using the aforementioned primers (Xavier et al., 2016) was used. In addition, we performed an *in silico* search of whole genome sequences with mlplasmids v. 1.0.0 (Arredondo-Alonso et al., 2018). To test whether the colistin resistance determinant was transferable, conjugation was performed by the broth filter mating method at 37°C using plasmid-free sodium azide resistant *E. coli* K12-J53 (J53 AziR) as recipient. Prior to the conjugation assays, all *mcr-2*-positive isolates were tested for their susceptibility to sodium azide. Transconjugants were selected on Endo agar plates containing 100 mg/L sodium azide and 2 mg/L colistin sulfate or containing 100 mg/L sodium azide and 4 mg/L colistin sulfate (Sigma-Aldrich, Germany, Karlsruhe, Germany). To confirm successful plasmid transfer, antimicrobial susceptibility testing of transconjugants, a PCR targeting the *mcr-2* gene and plasmid profiling was performed as described above.

### Plasmid analysis

To characterize the identified *mcr-2* gene harboring contigs, all contigs were aligned using Geneious (v. 8.1.9, Biomatters Ltd., Auckland, New Zealand) (Geneious, RRID:SCR_010519) to the respective gene. Contigs containing *mcr-2* were aligned to publicly available plasmid sequences from GenBank (GenBank, RRID:SCR_002760) using BLASTn analysis (RRID:SCR_004870). All contigs of a respective isolate were then aligned to the reference plasmid that revealed highest similarity. In addition, contigs were mapped to the selected reference plasmids using the Geneious Map to Reference. *In silico* constructed plasmids were further examined for mobile genetic elements, using ISfinder (RRID:SCR_003020). To display circular comparisons between plasmids we used the blast ring image generator software BRIG Version 0.95 (RRID:SCR_007802). Plasmid maps were generated using GenomeVx (Conant and Wolfe, 2008).

PlasmidFinder 2.1^5^ was applied to determine plasmid replicons. In addition, the assignment of MCR-2 plasmids to an incompatibility group was carried out by phylogenetic analysis of the sequences of the replication initiator protein TrfA and of plasmid “backbone” gene proteins TrfB, KlcA, KleA, KorC, TraD, TraE, TraF, TraG, TraJ, TraK, TraL, TrbA, TrbB, TrbC, TrbD, TrbF, TrgB, TrbI, TrbJ, and TrbK (Smith et al., 1993; Luppi et al., 2016).

### Phylogenetic comparison of *mcr-2* genes

To compare our *mcr-2* genes with currently known *mcr-2* genes, we screened the literature (PubMed, RRID:SCR_004846) and DNA sequence data repositories (GenBank, RRID:SCR_002760; EMBL, RRID:SCR_004473). Nucleotide and deduced amino acid sequences of *mcr-2* genes were downloaded from GenBank and aligned with Mafft v7.407 (RRID:SCR_011811). The resulting alignment was used to calculate a maximum likelihood-based phylogeny with RAxML v.8.2.10 (RRID:SCR_006086) with 100 bootstraps under the assumption of the gtr-gamma DNA substitution model (gamma BLOSUM62 protein substitution model).

### Detection of serotype and virulence-associated genes

The sero(geno)types of *E. coli* strains were determined using the web-based serotyping tool SerotypeFinder 2.0^6^ (Joensen et al., 2015). Screening for VAGs was carried out by NCBI BLASTn (RRID:SCR_004870) analysis against homologous genes present in an in-house database of 800 VAGs, gene variants or genomic islands from a subset of the VirulenceFinder database and in-house created and manually curated VAG reference sequences. We searched for genes that were previously linked with porcine intestinal pathogenic *E. coli* pathovars but also for VAGs of extraintestinal pathovars, such as uropathogenic *E. coli* (UPEC). Genes belonged to different categories (adhesin, toxin, iron uptake system, capsule synthesis, colicins, effector proteins, and secretion systems). Coverage length and sequence identity thresholds were 80% and 90%, respectively. *E. coli* isolates were further analyzed for the presence of the *fimH* gene and the allele type by aligning to a FimH database using FimTyper 1.0.^7^

### Phylogenetic grouping, multilocus sequence typing and core genome analysis

Phylogenetic groups were determined by using the ClermonTyping method and its associated web-interface ClermonTyper, that allows a given strain sequence to be assigned to *E. albertii, E. fergusonii*, *Escherichia* clades I-V, *E. coli sensu stricto* as well as to the seven main *E. coli* phylogroups A, B1, C, E, D, F, and B2 (Clermont et al., 2013; Beghain et al., 2018). MLST 2.0^8^ (Larsen et al., 2012) was applied to identify the multilocus sequence type (RRID:SCR_010245) of *E. coli* isolates following the Achtman scheme,^9^ which represents a 7-gene-scheme including genes *adk*, *fumC*, *gyrB*, *icd*, *mdh*, *purA*, and *recA*. Phylogenetic and population genetic relationships were determined by applying a gene-by-gene approach on the dataset to generate a core genome alignment and subsequently a phylogenetic tree. The core genome alignment was assembled by a gene-wise alignment (Mafft v7.407: RRID:SCR_011811) of 1,366 core genes that were present in at least 99% of the strains (sequence similarity min. 70%, sequence coverage min. 90%) and were concatenated afterward. The resulting alignment was used to infer a phylogeny with 100 bootstrap replicates using RAxML v.8.2.10 (RAxML, RRID:SCR_006086) with a General Time Reversible model and gamma correction for among site rate variation.

## Results

### Number and origin of *Escherichia coli* carrying *mcr-1* and *mcr-2* genes

Among the first collection of 7,614 *E. coli* isolates obtained from 2010 to 2017, 793 (10.4%) isolates were positive for *mcr-1* (Table 1). The rates of *mcr-1*-positive strains with respect to countries differed significantly, ranging from low (0–0.7%) (e.g., Switzerland, Austria, Netherlands, and Denmark) to moderate (8.8%–11.5%; Poland, Belgium and Germany) and high (33.3%–60.7%; Hungary, Spain, Italy and Portugal).

However, due to the biased sample material and the low number of *E. coli* isolates available from several countries, the data cannot be regarded as true prevalence data for most of the countries.

**FIGURE 1:**
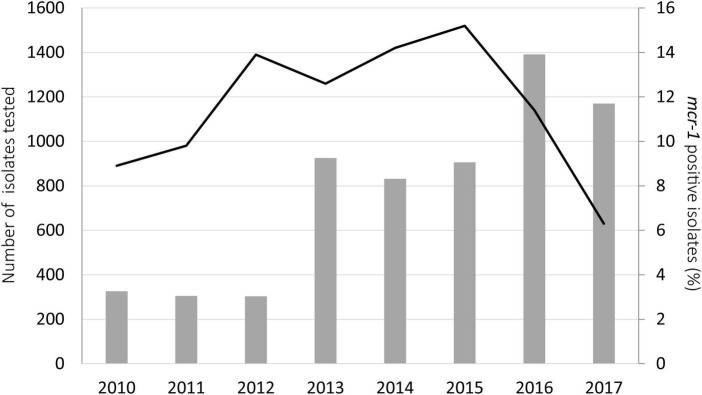
Annual rates of *mcr-1* (line) among 6,158 *E. coli* isolates (annual numbers as columns) collected from pigs in Germany from 2010 to 2017.

In Germany, where the highest number of samples was available, the rate of *mcr-1* among porcine *E. coli* isolates increased from 2010 (8.9%) to 2015 (15.2%), dropped slightly in the year 2016 (11.4%) and revealed the lowest percentage in 2017 (6.3%) (Figure 1, Supplementary Table 1).

In contrast to *mcr-1*, the *mcr-2* gene was only rarely detected in the first collection of 7,614 isolates. Twelve isolates were positive for *mcr-2*, representing 0.2% of the total number of isolates tested (Table 1). The majority of *mcr-2*-positive isolates was obtained from Belgium (9 isolates/within country rate of 8.0%), followed by Germany (2/0.03%) and Spain (1/3.6%). The Belgian isolates were all isolated in 2015 and originated from three different swine farms (farms I-III) (Table 2). The Spanish isolate dates back to October 2013 (farm IV) and the two strains from Germany were obtained in August and December 2014 on two different farms (farms V and VI).

**TABLE 2:** Characteristic features of *mcr-2*-positive *E. coli* isolates from pigs.

Strain	Month/Year of isolation	Country	Farm-pig-Isolate	MLST	cgMLST complex type	Phylo- group	Predicted serotype	Predicted pathotype	*mcr-2* gene	Plasmidome (>97.0% identity)**
IHIT31008*	02/2015	BE	I-1-1	ST100	12274	A	O138:H10	none	*mcr-2.1*	IncFII, IncFII(pSE11), IncI2, IncX1, IncX4
IHIT32395*	02/2015	BE	I-1-2	ST100	12274	A	O138:H10	ETEC	*mcr-2.1*	IncFIB(AP001918), IncFII, IncFII(pSE11), IncI2, IncX4
IHIT32396	02/2015	BE	I-2-1	ST100	12274	A	O138:H10	ETEC	*mcr-2.1*	IncFIB(AP001918), IncFII, IncFII(pSE11), IncI2, IncX4
IHIT32302	06/2015	BE	II-1-1	ST5786	3796	A	O182:H4	ETEC	*mcr-2.8*	IncFIB(AP001918), **IncX4**, IncFIA(HI1), IncHI1A, IncHI1B(R27), IncI1-I(Gamma), pO111
IHIT32399	06/2015	BE	II-2-1	ST5786	3796	A	O182:H4	ETEC	*mcr-2.8*	IncFIB(AP001918), **IncX4**, IncFIA(HI1), IncHI1A, IncHI1B(R27), IncI1-I(Gamma), pO111
IHIT32303*	06/2015	BE	II-3-1	ST3057	12275	clade I	O182:H4	ETEC-like	*mcr-2.1*	IncFIB(AP001918), **IncX4**, Col440II, IncFII, IncHI2, IncHI2A, IncY
IHIT32397	05/2015	BE	III-1-1	ST10	12276	A	O35:H6	ETEC	*mcr-2.1*	IncFIB(AP001918), **IncX4**, IncFII, IncI1-I(Gamma)
IHIT32305*	05/2015	BE	III-2-1	ST100	12277	A	O149:H10	ETEC	*mcr-2.1*	IncFIB(AP001918), **IncX4**, IncFIB(K), IncFIC(FII), IncFII(pSE11), IncI(Gamma), pO111
IHIT32304*	05/2015	BE	III-3-1	ST10	12276	A	O35:H6	ETEC-like	*mcr-2.1*	IncFIB(AP001918), **IncX4**, IncFII, IncI1-I(Gamma)
IHIT32403	10/2013	ES	IV-1-1	ST29	12280	B1	O45:H11	atypical EPEC	*mcr-2.1*	IncFIB(AP001918), Col(KPHS6), IncB/O/K/Z, IncFII, IncHI2, IndHI2A, IncX1
IHIT32401*	08/2014	DE	V-1-1	ST93	12278	A	O132:H25	ETEC-like	*mcr-2.1*	IncFIB(AP001918), **IncX4**, Col440II, IncFIA, IncHI2, IndHI2A, IncI1-I(Gamma)
IHIT32402*	12/2014	DE	VI-1-1	ST11875	12279	A	O132:Hnt	ETEC-like	*mcr-2.1*	IncFIB(AP001918), **IncX4**, IncFII, IncHI2, IncHI2A, IncY

BE, Belgium, DE, Germany, ES, Spain. *MCR-2 plasmids of these isolates could be successfully transferred to an *E. coli* K-12 recipient strain. **Plasmids that carry *mcr* genes are indicated with bold letters. The *mcr* gene of strain IHIT32403 is located on the chromosome; strains IHIT31008, IHIT32396, and IHIT32396 carry their *mcr* gene on an IncP-like plasmid.

Among the 1,477 *E. coli* isolates that we pre-screened on sheep blood agar containing 4 mg/L colistin, 64 isolates (4.3%) showed growth and were regarded as colistin non-susceptible (Col-non-S) isolates. Based on PCR analysis, none of the 64 Col-non-S isolates possessed *mcr-2*, while 36 isolates, i.e., 56.3% of Col-non-S strains and 2.4% from all 1,477 isolates tested, harbored the *mcr-1* gene (Table 1). All *mcr-1*-positive isolates were from Germany, accounting to 3.6% among 997 isolates tested in the years 2018-2020.

### Clonal relatedness of *mcr-2*-positive *Escherichia coli*

PFGE analyses separated the 12 *mcr-2*-positive *E. coli* isolates into eight different groups (A–H). Macrorestriction patterns revealed the clonality of three isolates from two pigs on farm I (pulsotype A) and of two isolates from two pigs from farm II (pulsotype B), respectively (data not shown). On farm II, *mcr-2*-positive isolates displayed different PFGE patterns (pulsotypes B and C), suggesting that they were not clonally related to those on farm I and III. Five different pulsotypes (A–E) were identified among the nine isolates from three farms in Belgium (*n* = 9). Isolates from Spain and Germany differed from Belgian isolates in their PFGE profiles (pulsotypes F–H). Among the total of eight PFGE profiles, seven different multilocus sequence types were determined (Table 2). We found ST100 in four isolates from Belgium collected on two different farms (farm I and III). On farm III, two ST10 isolates (IHIT323204 and IHIT32397) were additionally present. Sequence type ST5786, which is a single locus variant of ST10 (IHIT32302 and IHIT32399), and ST3057 (IHIT32303) were identified in isolates obtained from farm II. The isolate from Spain belonged to ST29 and the two German isolates were assigned to ST93 and a single locus variant of ST93 (ST11875) that differed in the *gyrB* allele (ST93: *gyrB*-6; ST11875: *gyrB*-405). With the exception of the ST29 isolate (phylogroup B1) and the ST3057 isolate (clade I), all *mcr-2*-positive isolates belonged to phylogenetic group A (Table 2).

### Antimicrobial susceptibility and antimicrobial resistance genes

Minimum inhibitory concentration (MIC) data for the 12 *mcr-2*-positive *E. coli* isolates are provided in Supplementary Table 2. All but one repeatedly tested isolate (IHIT32302, MIC 0.5 mg/L) were resistant to colistin (MICs 4 mg/L–16 mg/L). Additional resistances were determined for ampicillin (100%), piperacillin (100%), tetracycline (91.7%), trimethoprim/sulfamethoxazole (91.7%), and chloramphenicol (33.3%) (Table 3). Only one isolate (ST100-IHIT32305, Belgium) was resistant to the aminoglycosides gentamicin and tobramycin, which correlated with the presence of aminoglycoside acetyltransferase gene *aac(3)-IV* in this strain (Table 3). None of the isolates showed resistance to fluoroquinolone, third-generation cephalosporins or to carbapenems. The phenotypic antimicrobial resistance profile almost always corresponded with the presence of resistance genes in the respective isolates (Table 3).

**TABLE 3:** Antimicrobial susceptibility and resistance genes of *mcr-2*-positive *E. coli* isolates from pigs.

Strain	Phenotypic resistance	Antimicrobial resistance genes according to antibiotic classes*								
		BL	AMG	FQ	PMB	TET	FOL	PHE	ML	LIN
IHIT31008	AMP, PIP, TET, CHL, SXT, PMB, CST	*bla*_TEM–1B_	*aadA1*, *aadA10*, *aph(6)-Id*, *aph(3“)-Ib*	–	*mcr-2.1*	*tet*(B)	*sul1*, *dfrA1, dfrA14*	*catA1*	*mdf*(A)	*Inu*(G)
IHIT32395	AMP, PIP, TET, CHL, SXT, PMB, CST	*bla*_TEM–1B_	*aadA10*, *aph(6)-Id*, *aph(3“)-Ib*	–	*mcr-2.1*	*tet*(B)	*sul1*, *dfrA1, dfrA14*	*catA1*	*mdf*(A)	*Inu*(G)
IHIT32396	AMP, PIP, TET, CHL, SXT, PMB, CST	*bla*_TEM–1B_	*aadA1*, *aadA10*, *aph(6)-Id*, *aph(3“)-Ib*	–	*mcr-2.1*	*tet*(B)	*sul1*, *dfrA1, dfrA14*	*catA1*	*mdf*(A)	*Inu*(G)
IHIT32302	AMP, PIP, TET, SXT, PMB, CST	*bla*_TEM–1B_	*aadA1, aadA5, aph(6)-Id*, *aph(3“)-Ib*	GyrA S83L	*mcr-2.8*	*tet*(B)	*sul1, dfrA17*	–	*mdf*(A)	*Inu*(G)
IHIT32399	AMP, PIP, TET, SXT, PMB, CST	*bla*_TEM–1B_	*aadA1, aadA5, aph(6)-Id*, *aph(3“)-Ib*	GyrA S83L	*mcr-2.8*	*tet*(B)	*sul1, sul2, dfrA17*	–	*mdf*(A)	*Inu*(G)
IHIT32303	AMP, PIP, TET, PMB, CST	*bla*_TEM–1B_	*aadA2*	–	*mcr-2.1*	*tet*(B)	*sul1*	–	*mdf*(A)	–
IHIT32397	AMP, PIP, TET, SXT, PMB, CST	*bla*_TEM–1B_	*aadA1, aph(6)-Id*, *aph(3“)-Ib*	–	*mcr-2.1*	*tet*(A)	*sul1 sul2, dfrA1*	–	*mdf*(A)	–
IHIT32305	AMP, PIP, GEN, TOB, CHL, SXT, PMB, CST	*bla*_TEM–1C_	*aadA1*, *aadA2*, *aph(4)-Ia, aph(6)-Id*, *aph(3“)-Ib, aac(3)-IV*	GyrA S83L, ParC S80R	*mcr-2.1*		*sul2*, *sul3, dfrA12*	*cmlA1*	*mdf*(A)	–
IHIT32304	AMP, PIP, TET, SXT, PMB, CST	*bla*_TEM–1B_	*aadA1, aph(6)-Id*, *aph(3“)-Ib*	–	*mcr-2.1*	*tet*(A)	*sul1, dfrA1*	–	*mdf*(A)	–
IHIT32403	AMP, PIP, TET, SXT, PMB, CST	*bla*_TEM–1B_, *bla*_OXA–4_	*aadA2, ant(2“)Ia, aph*(3’)-Ia, *aph*(3’)-XV	–	*mcr-2.1*	*tet*(A), *tet*(M)	*sul1*, *sul2*, *sul3*, *dfrA1*, *dfrA12*	*cmlA1*, *catB3*	*mdf*(A), *mph*(E), *msr*(E)	–
IHIT32401	AMP, PIP, TET, SXT, PMB, CST	*bla*_TEM–1A_	*aadA1*, *aph(6)-Id*, *aph(3“)-Ib*	–	*mcr-2.1*	*tet*(B)	*sul1*, *sul2*, *dfrA1*	–	*mdf*(A), *mph*(B)	–
IHIT32402	AMP, PIP, TET, SXT, PMB, CST	*bla*_TEM–1B_	*aadA1*, *aph(6)-Id*, *aph(3“)-Ib*	–	*mcr-2.1*	*tet*(B)	*sul1, sul2, dfrA1*	–	*mdf*(A), *mph*(B)	–

*BL, beta-lactam, AMG, aminoglycoside, FQ, fluoroquinolone, PMB, polymyxin B, TET, tetracycline, FOL, folate pathway antagonist, PHE, phenicol, ML, macrolide, LIN, lincosamide.

### Virulence genes and serotypes of *mcr-2*-positive *Escherichia coli* isolates

Based on the presence of VAGs obtained from WGS data we conducted a pathotype prediction of the *mcr-2*-positive *E. coli* isolates. Enterotoxigenic *E. coli* (ETEC) are characterized by the presence of genes for heat labile (*eltA, eltB*) and/or stable (*esta, estb*) *E. coli* enterotoxins and genes for adhesive fimbriae (F4, *fae*; F5, *fan*; F6, *fas*; F17, *f17*; F18, *fed*). With a percentage of 83.3%, most of our isolates were classified as ETEC or ETEC-like (Table 2), as they harbored *estb* and/or *eltA*, often in combination with genes for F4 or F18 fimbriae, respectively (Supplementary Table 3). The ST29 *E. coli* isolate IHIT32403 represented an atypical enteropathogenic *E. coli* (aEPEC) as it harbored intimin gene *eae* and lacked bundle-forming pili adhesin genes *bfpA-L*, which together with *eae* are indicative for typical EPEC. Only one isolate, namely ST100-IHIT31008, could not be assigned to a recognized *E. coli* intestinal or extraintestinal pathotype. Apart from enterotoxin and ETEC fimbriae genes, all isolates possessed a number of additional VAGs related to adhesion, tissue damage, iron acquisition, secretion and serum resistance. In particular aEPEC strain IHIT32403 from Spain revealed a number of additional genes that are known to play a significant role in the pathogenesis of EPEC-induced disease, including adhesin genes *cfa, efa1, lpf*, and *paa* as well as type III secretion system and effector protein genes (*esp, sep, lifA, nle, tccP, and tir*) (Supplementary Table 3). Sero(geno)typing revealed the presence of serotypes O138:H10, O182:H4, O35:H6, O149:H10, and O132:H25/Hnt among ETEC and ETEC-like isolates and of serotype O45:H11 in the aEPEC isolate (Table 2).

### Identification of a novel *mcr-2* variant

**FIGURE 2:**
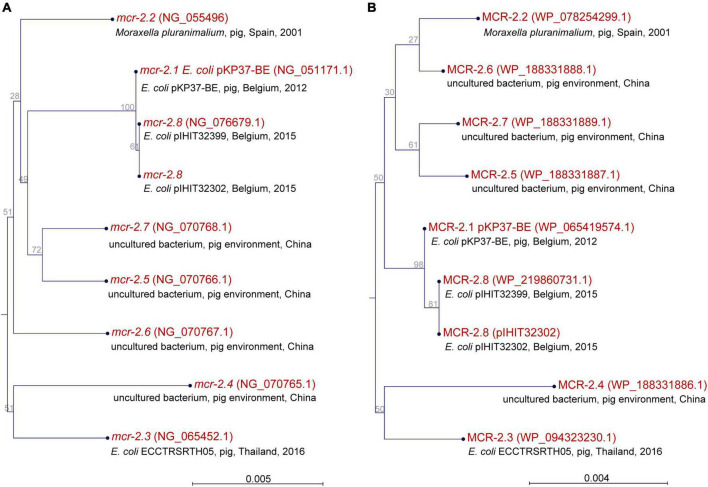
Phylogeny of seven confirmed and a novel *mcr-2* genes **(A)** and deduced MCR-2 proteins **(B)** based on a maximum-likelihood method.

At the time of writing, seven valid *mcr-2* genes (*mcr-2.1* to *mcr-2.7*) have been recognized. Gene *mcr-2.1* was discovered in *E. coli* isolates from pigs and cattle in Belgium (Xavier et al., 2016) and *mcr-2.2* was detected in *Moraxella pluranimalium* strain 248-01T (CCUG 54913) isolated from a pig in Spain in 2001 (Poirel et al., 2017). Another five genes, available in GenBank, were confirmed as reference sequences for *mcr-2* genes after curation of records by the NCBI staff (see Figure 2 for NCBI reference numbers). Nucleotide sequences obtained for the *mcr-2* genes (1,617 bp) of our *E. coli* isolates revealed that all but two isolates harbored *mcr-2.1* (Table 2). Two isolates from Belgium carried a novel variant of *mcr-2*, which had highest similarity (99.94%) to *mcr-2.1* from *E. coli* KP37-BE that harbored the originally described *mcr-2* gene (NG_051171). The novel *mcr-2* allele differed from *mcr-2.1* by encoding a single amino acid variation at position 390 (Met→Thr). Overall, *mcr-2.1* to *mcr-2.7* and our novel *mcr-2* allele had a similarity ranging from 94.19% (*mcr-2.4* vs. novel *mcr-2* from this study) to 99.94%. A comparison of the deduced amino acid sequences (538 aa) of *mcr-2* genes revealed similarity levels of 96.47% (MCR-2.4 vs. MCR-2.5) to 99.81% (MCR-2.1 vs. novel MCR-2 from this study). A maximum likelihood-based phylogeny of *mcr-2* gene alleles and MCR-2 proteins is provided in Figure 2.

### Genomic location and transferability of *mcr-2* genes

We identified *mcr-2* on two different plasmids (*n* = 11 isolates) and on the chromosome of our *E. coli* strains (*n* = 1 isolate). This was initially confirmed by S1 nuclease PFGE and Southern blotting (Supplementary Figure 1). Based on BLASTn analysis of constructed plasmid sequences, the MCR-2 plasmids of six *E. coli* isolates from Belgium and of the two German isolates were found to be highly similar (>99.8%) to the *mcr-2*-bearing IncX4 plasmid pKP37-BE (GenBank LT598652.1). Like pKP37-BE, they were 35 kb in size, belonged to incompatibility group IncX4, and possessed *mcr-2* as the sole resistance gene. An overview of plasmid incompatibility groups and estimated plasmid sizes of all *mcr-2*-positive *E. coli* found in this study is given in Supplementary Table 4.

**FIGURE 3:**
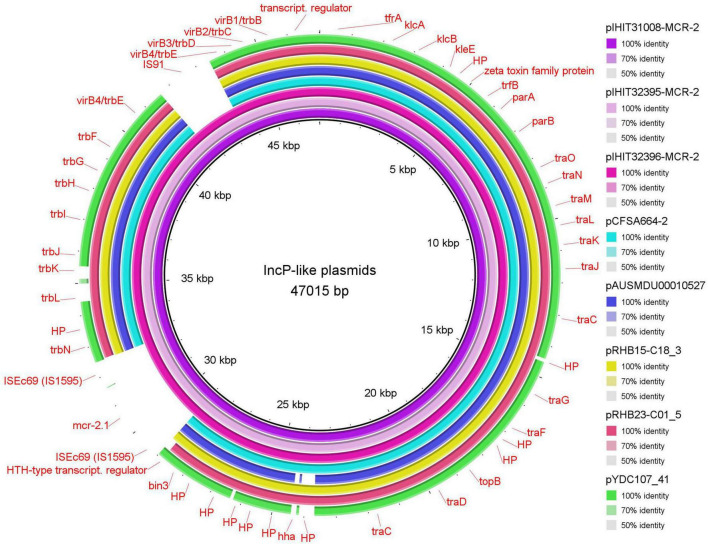
Circular representation of novel MCR-2 plasmids compared with five of the most similar reference plasmids available in GenBank (see Supplementary Table 4).

Three other isolates from Belgium revealed 47 kb MCR-2 plasmids (pIHIT31008-MCR-2, pIHIT32395-MCR-2, and pIHIT32396-MCR-2) that were identical in size and structure and also harbored *mcr-2* as the sole resistance gene (Table 2). The genetic organization of pIHIT31008-MCR-2 as a representative of these plasmids is shown in Supplementary Figure 2. BLASTn analysis of the pIHIT31008-MCR-2 nucleotide sequence against the GenBank database revealed that this plasmid shared conserved backbones with previously published plasmids of 41.4 to 46.5 kb in size that were identified in *E. coli*, *E. fergusonii* and *Salmonella* Enteritidis. Seven plasmids with highest query coverage (84.0%–88.0%) and query cover plasmid identity (96.95%–100%) to our plasmid are provided in Supplementary Table 5. They were identified in bacteria from food in China (*S. enterica*, pCFSA664-2), pooled sheep fecal samples in the UK (*E. coli*, pRHB15-C18_3; *E. fergusonii*, pRHB23-C01_5), pig feces in Canada (*E. coli*, plasmid unnamed_novel_0), and also from human patient samples collected in Australia (*S*. Enteritidis, pAUSMDU00010527), Japan (*E. coli*, pTHO-015-2), and the U.S.A. (*E. coli*, pYDC107_41) from 2008 to 2018. In contrast to our plasmid, the previous plasmids commonly lacked the *mcr-2*-containing cassette (IS*Ec69*-*mcr-2*-ORF-IS*Ec69*) and an insertion sequence element IS*91* that disrupted the type IV secretion system gene *virB4* genetic region on our 47 kb plasmids (Figure 3).

**FIGURE 4:**
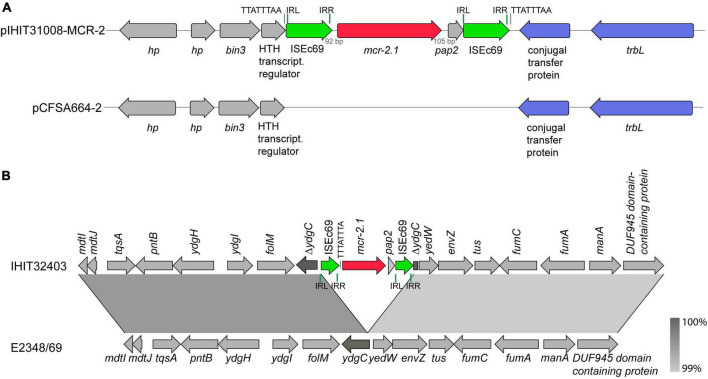
Genetic characterization of *mcr-2* genes. **(A)** Scheme of the integration site of the *mcr-2*-containing cassette into a plasmid backbone present in pCFSA664-2 (CP033354.2; *Salmonella enterica* ssp. *enterica* from food, China, 2015) and other plasmids, shown in Figure 3. **(B)** Linear comparison of a partial region (18,296 bp) of the genome of *mcr-2*-positive atypical EPEC strain IHIT32403 (diarrheic pig, Spain, 2013, ST29, O45:H11, phylogroup B1) and prototype EPEC strain E2348/69 (infantile diarrhea, UK, 1969, ST15, O45:H11, phylogroup B2; NZ_BDOY01.1). Boxed arrows represent the position and transcriptional direction of open reading frames. Insertion sequences are highlighted with green arrows, transfer genes with light blue arrows. Green vertical bars represent inverted regions (IR) at the left (IRL) and right site (IRR) of each IS element; black vertical bars represent 8-bp direct repeat sequences. Region of >99% identity are shaded in gray.

Figure 4A shows the integration site of the 3,489 bp *mcr-2* gene cassette into the common plasmid backbone region of the published plasmids. The *mcr-2* gene cassette of pIHIT31008-MCR-2 and of the two other 47 kb plasmids is almost identical (99.9%) to that described for pKP37-BE. Like in that IncX4 plasmid, the 1,617 bp *mcr-2* gene is flanked by directly oriented copies of insertion sequence element IS*Ec69* of the IS*1595* family (Partridge, 2017). We also identified a 297 bp open reading frame downstream of *mcr-2* on this element, which encodes a PAP2 membrane-associated lipid phosphatase with 41% identity to *Moraxella osloensis* phosphatidic acid phosphatase (71% query coverage).

The novel 47 kb MCR-2 plasmid was not predicted to have an Inc-type using PlasmidFinder. However, a BLASTn search of the IHIT31008 *trfA* gene, which encodes the initiation of plasmid replication, revealed four hits with a 100% nucleotide sequence identity to the IncP-1-like *trfA* replication genes of plasmids pAUSMDU00010527, pCFSA664-2, pRHB15-C18_3, and pRHB23-C01_5). In addition, the *trfA* genes of plasmids pYCD107, pTHO, and plasmid unnamednovel_0 showed 99.7% to 99.9% similarity to our genes (Supplementary Table 5).

A Neighbor-joining tree based on the alignment of TrfA amino acid sequences of representative members of the different IncP1 plasmid subfamilies α, β1, β2, δ, γ, ε, and ζ, our 47 kb MCR-2 plasmid and the aforementioned plasmids with identical backbones was created (Supplementary Figure 3). Interestingly, this showed that our MCR-2 plasmid and those with the shared backbone fall within the IncP1 family, but are phylogenetically different from the currently known IncP1 sub-families, indicating that they might represent a new subfamily. This clustering was also supported when performing a phylogeny inferred from concatenated sequences of the 22 plasmid backbone gene encoded proteins TrfA, TrfB, KfrA, KfrB, KlcA, KlcB, KorC, TraC, TraD, TraF, TraG, TraJ, TraK, TraL, TrbB, TrbC, TrbD, TrbF, TrbG, TrbI, TrbJ, and TrbK (data not shown) (Krol et al., 2012).

Seven out of eleven *mcr-2*-bearing plasmids from this study were successfully transferred to the recipient strain *E. coli* K12-J53. In case of strains IHIT32396 (47 kb IncP-1-like plasmid), IHIT32302, IHIT32399, and IHIT32397 (all IncX4 plasmids) conjugation experiments failed to transfer the pMCR-2 plasmid into the *E. coli* laboratory recipient. The seven pMCR-2 transformants showed a MIC of 4–8 mg/L for colistin, 16 to 32-fold higher than the empty J53 recipient (MIC = 0.25 mg/L).

The atypical EPEC isolate IHIT32403 carried its *mcr-2.1* gene on the chromosome. Attempts to transfer *mcr-2* by transconjugation failed, further confirming a chromosomal location of the gene. A linear comparison of a partial region (18,296 bp) of the genome of this ST29-B1 isolate of serotype O45:H11, that was obtained from a diarrheic pig in Spain in 2013, and of prototype O45:H11-ST15-B2 EPEC strain E2348/69, obtained from infantile diarrhea in Great Britain in 1969 (NZ_BDOY01.1) is shown in Figure 4B. The two regions were 100% identical, except for the insertion of gene cassette IS*Ec69*-*mcr-2*-*pap2*-IS*Ec69* into the inner membrane protein gene *ydgC* in isolate IHIT32403.

## Discussion

The colistin resistance gene *mcr-1* gene is disseminated in many countries on all continents except Antarctica, and has been reported from various Gram-negative bacterial species from animals, human and the environment, such as *E. coli*, *Klebsiella* spp., *Salmonella enterica* serovars, *Enterobacter* spp. and *Moraxella* spp. (Nang et al., 2019; Anyanwu et al., 2021; Valiakos and Kapna, 2021). Our study revealed a prevalence of 11.5% *mcr-1*-positive isolates among pathogenic *E. coli* isolates collected from German pig farms in 2010-2017. A retrospective study on preselected bacterial cultures from asymptomatic fattening pigs, isolated in 2011 and 2012 in Germany, showed a similar prevalence of 9.9% (Roschanski et al., 2017). In a more recent study, Effelsberg et al. (2021) investigated the prevalence of mobile colistin resistance genes *mcr-1* to *mcr-5* in *Enterobacterales* from 81 pig farms in North-West Germany. In that study, the authors included fecal samples from pigs and stool samples from directly exposed humans working on the respective farms in 2018-2020. Two (1.4%) out of 138 stool samples from farmers, farm workers and their family members were tested positive for *mcr-1*, though a direct transmission could not be verified. The *mcr-1* gene was detected in 5.7% of the porcine samples (Effelsberg et al., 2021).

Considering the overall rate of *mcr-1* determined in the present study, including isolates from other European countries, prevalences were 10.4% in 2010-2017 and 2.4% in 2018-2020. From 2018 to 2020 we used selective culturing of bacteria on sheep blood agar containing colistin. Therefore, we cannot not exclude that some isolates carrying the *mcr-1* gene were missed. Thus, no conclusions on prevalence dynamics can be drawn from comparison of these two data, but noteworthy, annual rates of *mcr-1*-positive *E. coli* isolates started to decrease already in 2016. Similar results were reported from Spain. Miguela-Villoldo et al. described a steady increase of *mcr-1* in bacteria from cecal samples from pigs between 2004 and 2015 and a downward trend between 2017 and 2021 (Miguela-Villoldo et al., 2022). Another group from Spain screened 200 *E. coli* isolates collected in 1999 to 2018 from swine and reported a peak of colistin resistance (17.5% of the strains) in 2011–2014 (Aguirre et al., 2020). Our observation that *mcr-1* occurrence is much less frequent in samples collected after 2015 is in line with the studies reported.

It needs to be verified whether observed decreases are already a consequence of the restrictions recommended by the WHO for the use of important antimicrobials, including colistin (WHO, 2017). Indeed, an observational study from Great Britain revealed that the stoppage of colistin usage in pig farms could lead to a lower persistence of mobile colistin resistance genes on a long-term (Duggett et al., 2018). Based on data from longitudinal studies performed in China, authors could determine an association between significantly reduced colistin sales and a decreased frequency of *mcr-1*-producing *E. coli* in pig feces and in the intestinal tract of healthy humans as well as decreased rates of human infections with colistin resistant *E. coli* after 2017. It was suggested that this might be a direct consequence of banning colistin as an animal growth promoter by the Chinese government in April 2017 (Wang et al., 2020b; Zhao et al., 2022). On the other hand, no significant association could be found in a cross-sectional study on 48 pig farms in 2011-2012 between presence of *mcr-1* resistance genes in isolated *E. coli* and antimicrobial treatment of the sampled fattening pigs (Hille et al., 2018). In another study, healthy weaned piglets were inoculated with a colistin-resistant *E. coli* strain harboring *mcr-1*. Following this, one group of piglets was force-fed with colistin sulfate for five consecutive days. The double dose of colistin sulfate was given to a second group of piglets and a placebo group received water over the same period of time. A selection of *mcr*-positive strains was not observed, as the prevalence of the inoculated *mcr-1*-positive *E. coli* remained almost at the same levels in all groups over 25 days (Viel et al., 2018).

The *mcr-2* gene, which was detected soon after *mcr-1*, was globally found on rare occasions and predominantly in Asian countries, including Bangladesh, China, India, Pakistan, Thailand, and Turkey (Valiakos and Kapna, 2021). Nine out of 40 studies performed in Europe reported infrequent to moderate findings of *mcr-2* (0.15% – 11.4%) in samples, isolates or in the microbiota of livestock animals in Great Britain, Spain, Italy, and especially in Belgium in the years 2001 to 2019 (Xavier et al., 2016; AbuOun et al., 2017; Carattoli et al., 2017; Poirel et al., 2017; Garcia-Graells et al., 2018; Dobrzanska et al., 2020; Miguela-Villoldo et al., 2020; Bertelloni et al., 2022) (Supplementary Table 6). Although we tested 9,091 *E. coli* isolates from Germany and other European countries, only 12 isolates were *mcr-2*-positive. Out of these, nine isolates were obtained from three different pig farms in Belgium, which underlines the local spread of *mcr-2* in this country. Interestingly, the two *mcr-2*-positive *E. coli* isolates from two different pig farms in Germany, collected in 2014, are, to the best of our knowledge, the first reported *mcr-2* findings in this country. In two studies from 2011/2012 and 2011-2018 healthy animals from Germany, in particular pigs, were sampled and Gram-negative bacteria were tested for the presence of *mcr-1* to *mcr-2* and *mcr-1* to *mcr-9*, respectively. While 9.9% of the 436 tested mixed bacterial cultures and 45% of 407 tested *Salmonella enterica* isolates were *mcr-1*-positive, none of the cultures and isolates harbored *mcr-2* (Roschanski et al., 2017; Borowiak et al., 2020). In 2016 and 2017, raw municipal wastewater was sampled all over Germany and analyzed in a metagenomics approach. Genes *mcr-3*, *mcr-4*, *mcr-5*, and *mcr-7* were ubiquitous in all 14 samples, but only one proved positive for *mcr-1* and none for *mcr-2* (Kneis et al., 2019). Likewise, *mcr-2* was not detected in a very recent study performed on 456 samples obtained from pigs and humans in Germany, where all isolated *Enterobacterales* were tested for the genes *mcr-1* to *mcr-5* (Effelsberg et al., 2021). The systematic review by Valiakos et al. reported *mcr-2* in five studies from swine, three from bovine and three from poultry (Valiakos and Kapna, 2021). Total numbers of *mcr-2* were low, in particular in Europe and Africa. The vast majority of *mcr-2*-positive bacteria in that survey were *E. coli*. An exception was one *Moraxella pluranimalium*-like isolate harboring a *mcr-2.2* variant isolated from pooled cecal samples of healthy pigs (AbuOun et al., 2017). A more recent study from Egypt tested *Enterobacterales* isolates, collected between 2018 and 2020 from bovine milk samples associated with mastitis for the genes *mcr-1* to *mcr-9*. Among 117 tested isolates, 12.0% possessed *mcr-2* and another 28.2% carried *mcr-1*, *mcr-3*, *mcr-4* or *mcr-7* genes (Tartor et al., 2021).

In non-European countries *mcr-2* mediated colistin resistance was systematically tested in 64 studies (Supplementary Table 6). Twenty-five studies (38.5%) found bacteria harboring *mcr-2* resistance genes. Peak prevalences were as high as 56.3% in healthy pigs in 2016 and 14.9% in healthy chickens in 2015-2016 in China (Zhang et al., 2018b; Zhang et al., 2019).

Also wild animals, reptiles and pets have been identified as a source of *mcr-2*-positive bacteria. In 2018 and 2019, 26/168 (15.5%) *E. coli* isolates from hunted wild boar harbored *mcr-2* (Cilia et al., 2021). A study from Egypt identified *mcr-2* in 2.5% of 122 Gram-negative bacterial isolates from wild birds (Ahmed et al., 2019). In pet animals, the prevalence of *mcr-1* ranged from 0% to 12.5%, that of *mcr-2* from 0% to 0.9% (Borowiak et al., 2020; Ilbeigi et al., 2021; Wang et al., 2021; Bertelloni et al., 2022; Hamame et al., 2022).

In 2018, Partridge et al. (2018) proposed a nomenclature scheme for *mcr* genes and their variants, therefore systematizing future registration of new discoveries of genes and allele numbers (Partridge et al., 2018). Their scheme suggested pre-publication submission of new *mcr* sequences to the International Nucleotide Sequence Database Collaboration.^10^ New *mcr* gene variants are defined by deduced amino acid sequence and assigned by the National Center for Biotechnology Information (NCBI). To date, a regularly updated overview of known *mcr* genes and variants is available in the Bacterial Antimicrobial Resistance Reference Gene Database, maintained by the NCBI.^11^ So far, seven genetically different *mcr-2* variants are described in the literature (*mcr-2.1* – *mcr-2.7*). We here report an additional genetic variant, termed *mcr-2.8*, which is closely related to *mcr-2.1* and was found in *E. coli* isolates from pigs in Belgium. All other *mcr-2* genes in our samples were *mcr-2.1* (Table 2), which is also the most frequent variant worldwide.

Out of 12 *mcr-2*-positive bacterial isolates, ten could be typed as enterotoxigenic *E. coli* (ETEC) or ETEC-like and one as an atypical enteropathogenic *E. coli* (aEPEC). Another isolate from Belgium could not be grouped to any known *E. coli* pathotype as it lacked all taxonomical relevant combinations of virulence genes. ETEC are known for their frequent association with profuse neonatal and post-weaning diarrhea in pigs and calves (Foster and Smith, 2009; Liu et al., 2014). In an epidemiological study from 2018, ETEC was prevalent in 67% of post-weaning piglets suffering from diarrhea, followed by aEPEC (21.7%) (Garcia-Menino et al., 2018). In the same study, aEPEC was the most commonly detected pathovar (60.3%) among samples from diarrheic suckling piglets. The Global Burden of Disease study, performed between 1990 and 2016 in the USA, analyzed the impact of human diarrhea caused by ETEC on social and economic factors of health. At all age groups, ETEC was responsible for about 3.2% of deaths due to diarrhea in 2016 (Khalil et al., 2018). In humans, aEPEC are also regarded as important bacterial pathogens that are particularly involved in persistent diarrhea in children under five years of age (Afset et al., 2004; Mora et al., 2016; Snehaa et al., 2021). Unfortunately, the occurrence of *mcr* genes was not tested in the studies reporting on human ETEC and aEPEC isolates. Therefore, it remains to be determined whether *mcr-*mediated colistin resistance is relevant in *E. coli* strains causing diarrhea in humans.

Six *mcr-2* genes and the two novel *mcr-2* variants were found on IncX4 plasmids, which are known to play a vital role in the distribution of *mcr-1* genes among *Enterobacterales* (Doumith et al., 2016; Jamin et al., 2021; Abou Fayad et al., 2022; Xie et al., 2022). The IncX4 plasmids detected in our study showed high sequence similarity (>99.8%) to the originally described plasmid pKP37-BE from *E. coli* in 2011/2012 (Xavier et al., 2016). This is in line with previously reported occurrences of pKP37-BE-like plasmids, indicating a steady appearance of this plasmid in Belgium in association with *mcr-2* (Garcia-Graells et al., 2018; Timmermans et al., 2021). Our findings support the idea that IncX4 plasmids also play a role in the spread of *mcr-2* genes.

So far, the only plasmid groups identified in association with *mcr-2* were IncX4 and IncHI1B/IncFIB (Stosic et al., 2021). We found one new *mcr-2*-harboring plasmid (IncP-like plasmid), which was detected in three pigs on the same farm in Belgium. Plasmid transferability was observed in two of three *E. coli* isolates. This newly described plasmid represents to the best of our knowledge the third plasmid group beside IncX4 and IncHI1B/IncFIB harboring the *mcr-2* gene. The low frequency of *mcr-2-*IncP-like plasmids in our strain collection suggests that this plasmid type has rarely spread yet. However, only 9 of 39 studies mentioned in Supplementary Table 6 explored the genomic location of *mcr-2* and characterized the plasmids in more detail (Xavier et al., 2016; AbuOun et al., 2017; Poirel et al., 2017; Garcia-Graells et al., 2018; Stosic et al., 2021; Tartor et al., 2021; Timmermans et al., 2021; Trongjit and Chuanchuen, 2021; Phuadraksa et al., 2022). Thus, occurrence of IncP-like plasmids carrying the *mcr-2* gene may be underestimated.

This study has some limitations. We have few information about pig housing conditions, hygiene standards, previous antimicrobial treatment and animal trafficking. Due to differing numbers of sample submissions from the different European countries throughout the years, the collected data are not representative of the target population. At the time of writing, ten different *mcr* genes (*mcr-1* to *mcr-10*) were reported. In this study, we concentrated on mobilizable colistin resistant genes *mcr-1* and *mcr-2*. Thus, future investigations on the distribution of the remaining *mcr* genes among the strain collection would be preferable. The chosen laboratory method since 2018, i.e., selective culturing of isolates on sheep blood agar containing 4 mg/L colistin, may have led to a fewer detection rate of *mcr*-positive isolates with low or diminished growth rates under these conditions (Smelikova et al., 2022).

On the other hand, our study has several strengths. We performed an extensive screening of plasmid-mediated colistin resistance genes (*mcr-1* and *mcr-2*) on a large pool of putative pathogenic *E. coli* collected from pigs over a longer period of time. Moreover, *mcr-2*-positive isolates and their plasmids underwent detailed genotyping, while also the transferability of *mcr-2* genes was explored.

## Conclusion

We could confirm a continuous and substantial decrease in the percentage of porcine *E. coli* isolates harboring *mcr-1* from 2015 to 2017. As expected, *mcr-2*-mediated colistin resistance was much less frequent than that conferred by *mcr-1*. We here described the new *mcr-2.8* variant and a new *mcr-2.1*-bearing IncP1-like plasmid, which is capable of transferring colistin resistance to susceptible *E. coli* by conjugation. These novel variants and recent reports of *mcr-2*-positive bacteria in previously less frequently tested samples like bovine milk, pet animals and reptiles suggest that colistin resistance genes are highly variable and represent a constant threat to animal and human welfare. There is an urgent need for a longitudinal monitoring of *mcr* genes in Gram-negative bacteria from different sectors, including humans, animals, food products and the environment. This would ensure that the distribution of colistin resistant strains and putative novel resistance mechanisms are detected at an early stage. Gained knowledge about the distribution and epidemiology of *mcr* genes might help to prevent epidemic occurrence of multidrug resistant bacteria.

## Data availability statement

Read genome sequences used in this study were submitted to the Sequence Read Archive (SRA) platform available from NCBI (https://trace.ncbi.nlm.nih.gov/Traces/sra) under biosample accession numbers: SAMN17614371 – SAMN17614382 and Sequence Read Run (SRR) numbers: SRR13570475 – SRR13570486.

## Author contributions

CE and RB supervised the entire project. CE, LG, and RB drafted the manuscript. CE, KK, and RB designed the study. EP-B, CE, TS, and LG have provided raw data and/or analyzed the data and conducted part of the laboratory experiments. CE, TS, and LG performed analyses of sequencing data. All authors critically reviewed the manuscript.

## Footnotes

1
https://www.ncbi.nlm.nih.gov/pathogens/isolates#/refgene/

2
http://www.eucast.org

3
https://cge.food.dtu.dk/services/ResFinder/

4
https://card.mcmaster.ca

5
https://cge.food.dtu.dk/services/PlasmidFinder/

6
https://cge.food.dtu.dk/services/SerotypeFinder/

7
https://cge.food.dtu.dk/services/FimTyper/

8
https://cge.food.dtu.dk/services/MLST/

9
https://pubmlst.org/mlst/

10
https://www.insdc.org/

11
https://www.ncbi.nlm.nih.gov/pathogens/refgene/#mcr
