# Supplementary material for: Occurrence of mcr-1 and mcr-2 colistin resistance genes in porcine Escherichia coli isolates (2010–2020) and genomic characterization of mcr-2-positive E. coli
Source: Front Microbiol. 2022 Dec 9;13:1076315. doi: 10.3389/fmicb.2022.1076315 (PMC9780603; doi:10.3389/fmicb.2022.1076315)
Supplement: Supplementary file 1 [file Image_1.pdf]

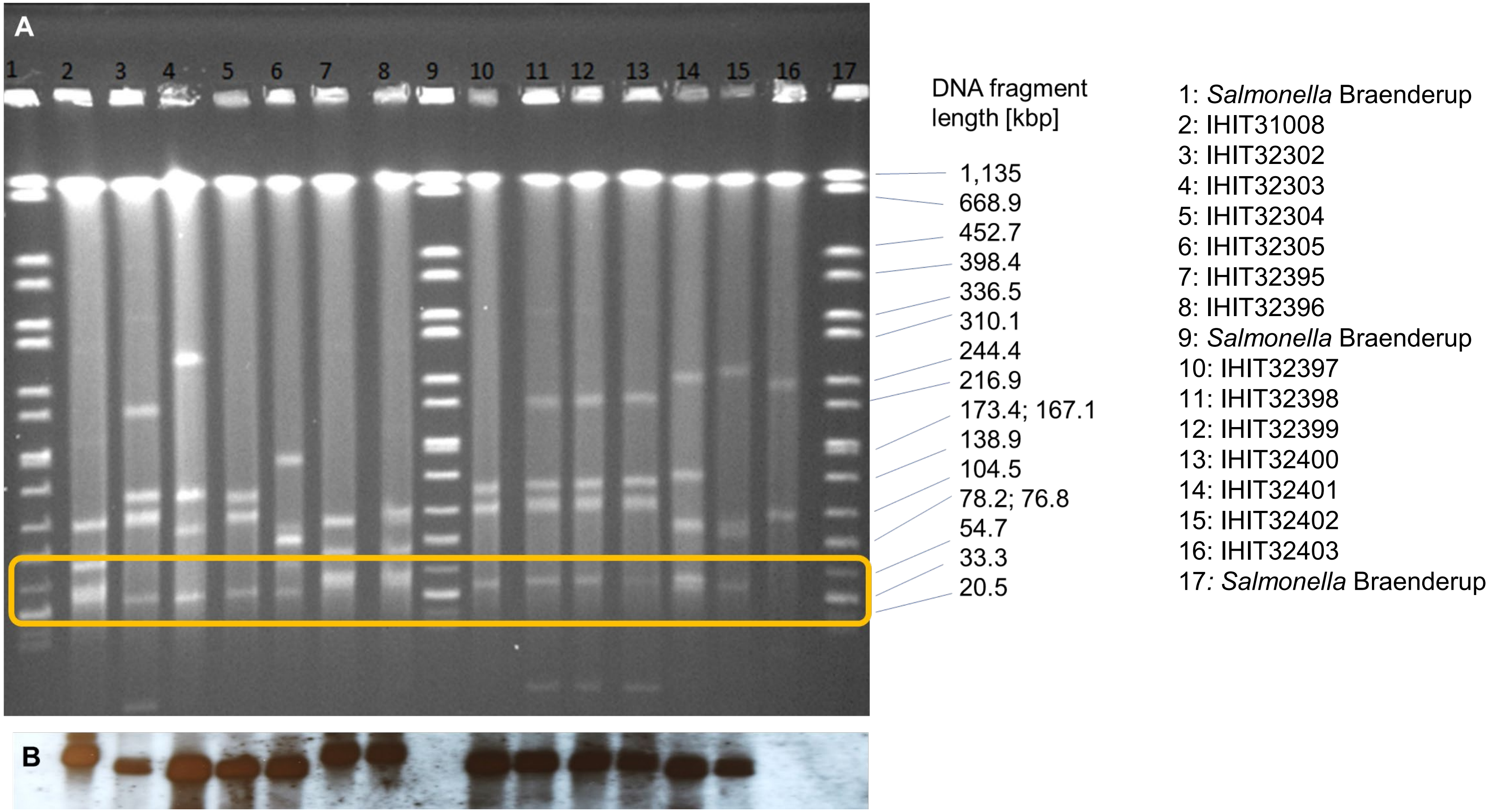

Supplementary Figure 1: S1-nuclease digested genomic DNA obtained from 14 *mcr-2*-positive *E. coli* isolates (IHIT32398 and IHIT32400 were duplicates to IHIT32399; thus, they were not included in the main text). **A** PFGE electropherogramm; **B** Southern blot after hybridization with *mcr-2* probe (the blot section corresponds to the area highlighted in Fig. A).
