# Supplementary material for: Occurrence of mcr-1 and mcr-2 colistin resistance genes in porcine Escherichia coli isolates (2010–2020) and genomic characterization of mcr-2-positive E. coli
Source: Front Microbiol. 2022 Dec 9;13:1076315. doi: 10.3389/fmicb.2022.1076315 (PMC9780603; doi:10.3389/fmicb.2022.1076315)
Supplement: Supplementary file 2 [file Image_2.pdf]

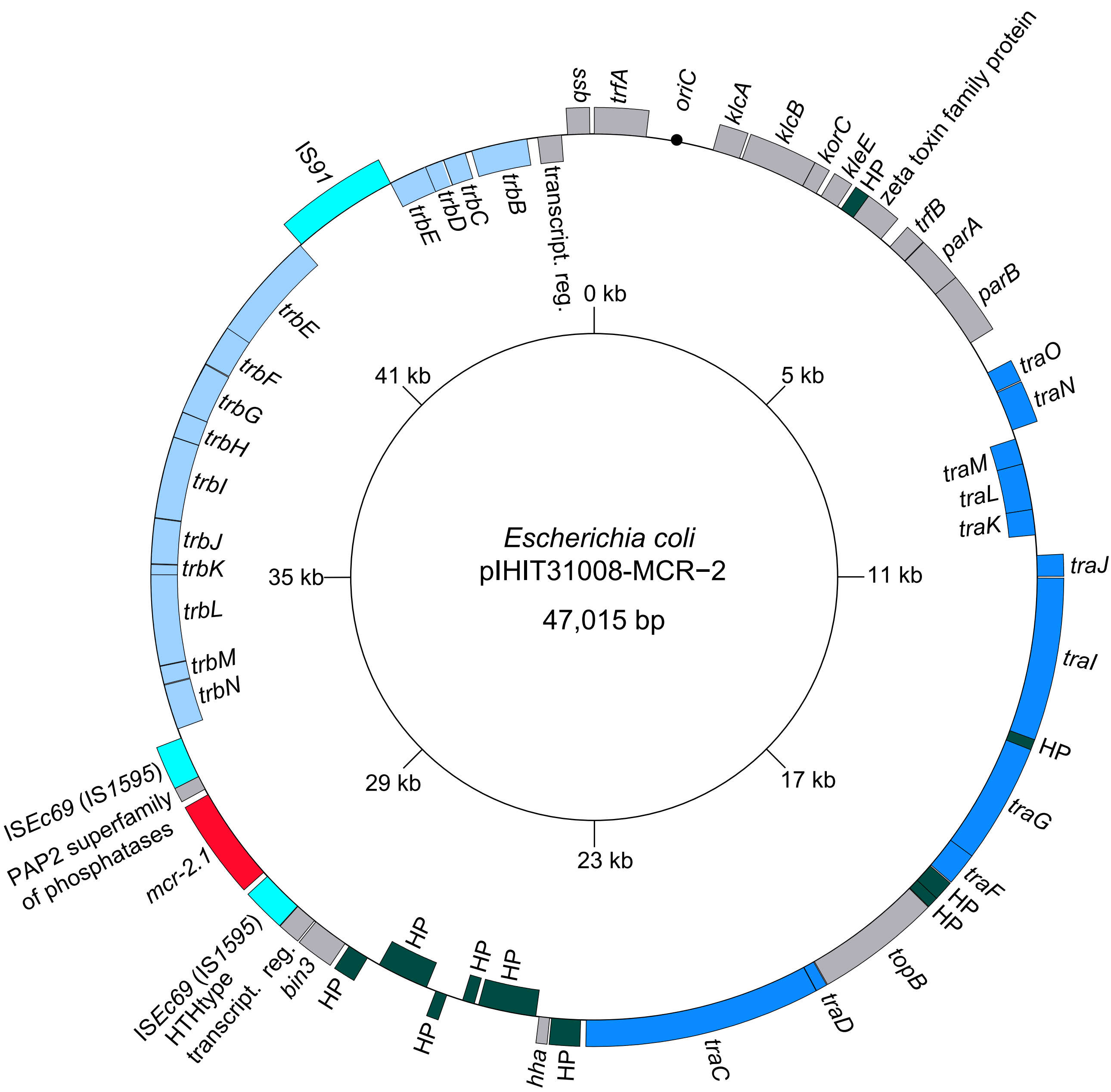

Supplementary Figure 2: Genetic organisation and structure of *mcr-2.1* harbouring plasmid pIHIT31008-MCR-2 from colistin-resistant porcine *Escherichia coli* isolate IHIT31008.
