# Supplementary figures and images for: Occurrence of mcr-1 and mcr-2 colistin resistance genes in porcine Escherichia coli isolates (2010–2020) and genomic characterization of mcr-2-positive E. coli
Source: Front Microbiol. 2022 Dec 9;13:1076315. doi: 10.3389/fmicb.2022.1076315 (PMC9780603; doi:10.3389/fmicb.2022.1076315)

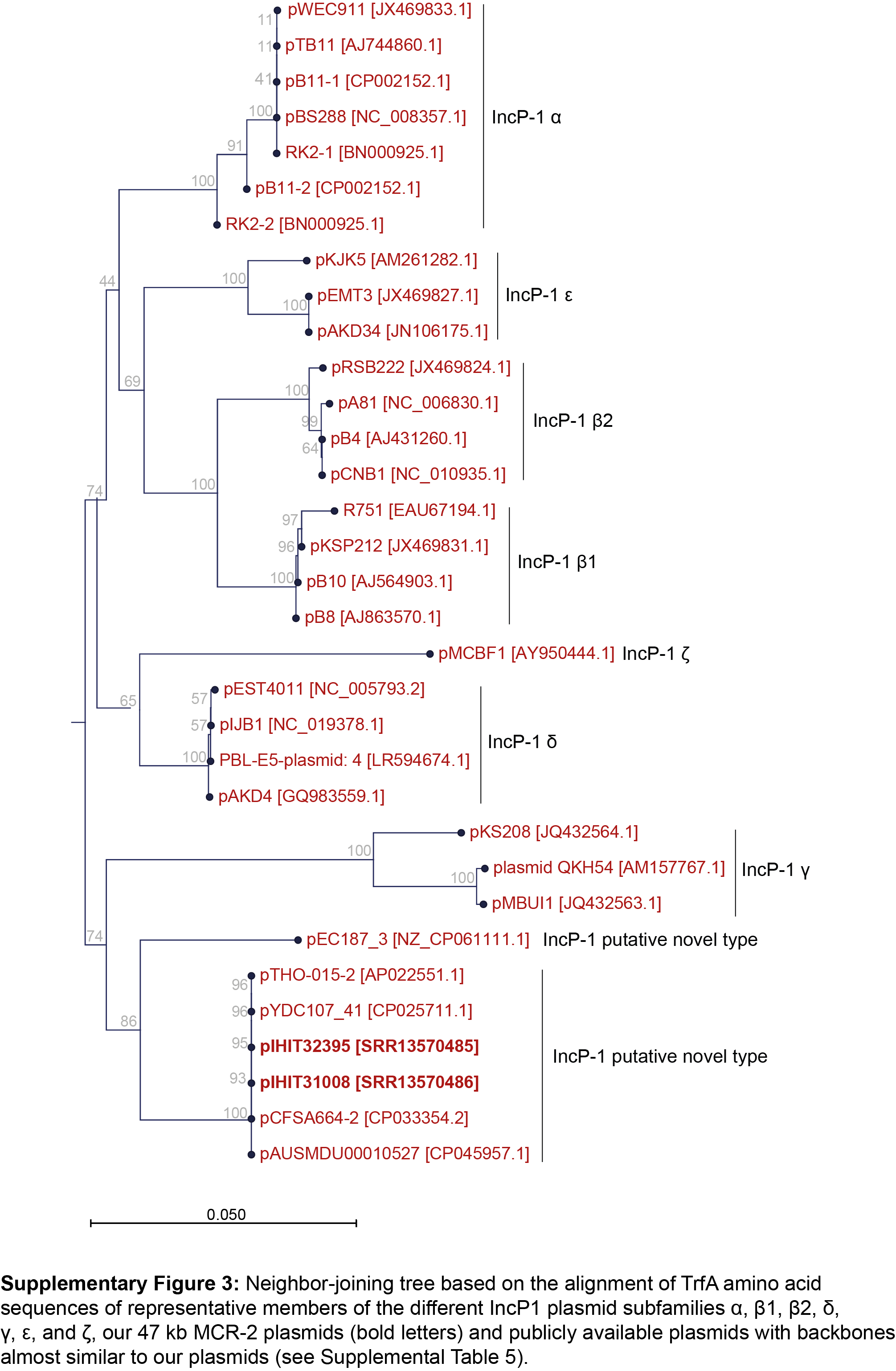

Supplement: Supplementary file 3 [file Image_3.png]
