# Supplementary material for: Occurrence of mcr-1 and mcr-2 colistin resistance genes in porcine Escherichia coli isolates (2010–2020) and genomic characterization of mcr-2-positive E. coli
Source: Front Microbiol. 2022 Dec 9;13:1076315. doi: 10.3389/fmicb.2022.1076315 (PMC9780603; doi:10.3389/fmicb.2022.1076315)
Supplement: Supplementary file 4 [file Data_Sheet_1.pdf]

Supplementary Tables

Supplementary Table 1:
Annual rates of *mcr-1* and *mcr-2* among porcine *E. coli* isolates collected between 2010 and 2017

Supplementary Table 2:
MIC data for *mcr-2* positive *E. coli* isolates from pigs

Supplementary Table 3:
Virulence-associated genes (90% coverage, ≥ 90% identity) in *mcr-2* positive *E. coli* isolates and predicted pathotype

Supplementary Table 4:
Plasmid incompatibility groups and estimated plasmid sizes of *mcr-2* positive *E. coli* from pigs

Supplementary Table 5:
Characteristics of plasmids that share a common backbone to the novel 47-kb MCR-2 plasmid of porcine *E. coli* IHIT31008

Supplementary Table 6:
Occurrence of *mcr-2* and other *mcr* genes in samples from pigs and other animals, from animal meat, humans and the environment according to 104 publications. Only studies that included a screening for the *mcr-2* gene were considered.

Supplementary Table 1: Annual rates of *mcr-1* and *mcr-2* among porcine *E. coli* isolates collected between 2010 and 2017

**Country**	**2010-2017**				
	**Isolates**	***mcr-1***		***mcr-2***	
	**n**	**n**	**%**	**n**	**%**
**All countries**					
2010	392	29	7.4	0	0
2011	349	30	8.6	0	0
2012	346	42	12.1	0	0
2013	1,006	142	14.1	1	0.1
2014	1,179	134	11.4	2	0.2
2015	1,315	165	12.5	9	0.7
2016	1,612	165	10.2	0	0
2017	1,415	86	6.1	0	0
	**7,614**	**793**	**10.4**	**12**	**0.2**
**Germany (total)**					
2010	326	29	8.9	0	0
2011	305	30	9.8	0	0
2012	303	42	13.9	0	0
2013	925	117	12.6	0	0
2014	832	118	14.2	2	0.2
2015	906	138	15.2	0	0
2016	1,391	159	11.4	0	0
2017	1,170	74	6.3	0	0
	**6,158**	**707**	**11.5**	**2**	**0.03**
**Northern Germany***					
2010	62	2	3.2	0	0
2011	39	2	5.1	0	0
2012	30	7	23.3	0	0
2013	216	17	7.9	0	0
2014	222	46	20.7	1	0.5
2015	233	29	12.4	0	0
2016	438	51	11.6	0	0
2017	260	19	7.3	0	0
	**1,500**	**173**	**11.5**	**1**	**0.07**
**Western Germany***					
2010	136	24	17.6	0	0
2011	153	20	13.1	0	0
2012	105	6	5.7	0	0
2013	178	20	11.2	0	0
2014	194	27	13.9	1	0.5
2015	289	45	15.6	0	0
2016	387	45	11.6	0	0
2017	313	22	7.0	0	0

Country	2010-2017				
	**Isolates**	***mcr-1***		***mcr-2***	
	**n**	**n**	**%**	**n**	**%**
	**1,755**	**209**	**11.9**	**1**	**0.06**
**Eastern Germany***					
2010	40	1	2.5	0	0
2011	27	6	22.2	0	0
2012	38	8	21.1	0	0
2013	64	6	9.4	0	0
2014	282	37	13.1	0	0
2015	149	42	28.2	0	0
2016	241	36	14.9	0	0
2017	253	23	9.1	0	0
	**1,094**	**159**	**14.5**	**0**	**0**
**Southern Germany***					
2010	88	2	2.3	0	0
2011	86	2	2.3	0	0
2012	130	21	16.2	0	0
2013	467	74	15.8	0	0
2014	134	8	5.9	0	0
2015	235	22	9.4	0	0
2016	325	27	8.3	0	0
2017	344	10	2.9	0	0
	**1,809**	**166**	**9.2**	**0**	**0**

*Northern Germany: including Federal States Schleswig-Holstein, Hamburg, Lower Saxony and Bremen; Western Germany: including Federal States North Rhine-Westphalia, Hesse, Rhineland-Palatinate and Saarland; Eastern Germany: including Federal States Mecklenburg-Vorpommern, Berlin, Brandenburg, Saxony, Saxony-Anhalt and Thuringia; Southern Germany: including Federal States Bavaria and Baden-Württemberg

Supplementary Table 2: MIC data for *mcr-2* positive *E. coli* isolates from pigs

Strain-ID	AMP	AMC	PIP	LEX	CPD	CFT	CEP	IPM	AMK	GEN	TOB	ENR	MAR	TET	NIT	CHL	PMB	CST*	SXT
**IHIT31008**	≥ 32	4	≥ 128	8	≤ 0,25	≤ 1	≤ 1	≤ 1	≤ 2	≤ 1	≤ 1	≤ 0,12	≤ 0,5	≥ 16	64	≥ 64	8	4	≥ 3/20
**IHIT32395**	≥ 32	4	64	8	≤ 0,25	≤ 1	≤ 1	≤ 1	≤ 2	≤ 1	≤ 1	≤ 0,12	≤ 0,5	≥ 16	64	≥ 64	≥ 16	16	≥ 3/20
**IHIT32396**	≥ 32	4	≥ 128	8	≤ 0,25	≤ 1	≤ 1	≤ 1	≤ 2	≤ 1	≤ 1	≤ 0,12	≤ 0,5	≥ 16	64	≥ 64	≥ 16	16	≥ 3/20
**IHIT32302**	≥ 32	4	≥ 128	8	0,5	≤ 1	≤ 1	≤ 1	≤ 2	≤ 1	≤ 1	0,5	≤ 0,5	≥ 16	≤ 16	4	0,5	0,5	≥ 3/20
**IHIT32399**	≥ 32	4	≥ 128	8	0,5	≤ 1	≤ 1	≤ 1	≤ 2	≤ 1	≤ 1	1	1	≥ 16	≤ 16	8	8	4	≥ 3/20
**IHIT32303**	≥ 32	4	≥ 128	8	≤ 0,25	≤ 1	≤ 1	≤ 1	≤ 2	≤ 1	≤ 1	≤ 0,12	≤ 0,5	≥ 16	≤ 16	4	8	8	≤ 20
**IHIT32397**	≥ 32	4	≥ 128	8	≤ 0,25	≤ 1	≤ 1	≤ 1	≤ 2	≤ 1	≤ 1	≤ 0,12	≤ 0,5	≥ 16	32	≤ 2	8	4	≥ 3/20
**IHIT32305**	≥ 32	16	≥ 128	8	≤ 0,25	≤ 1	≤ 1	≤ 1	≤ 2	≥ 16	≥ 16	1	1	≤ 1	≤ 16	32	≥ 16	8	≥ 3/20
**IHIT32304**	≥ 32	4	64	8	≤ 0,25	≤ 1	≤ 1	≤ 1	≤ 2	≤ 1	≤ 1	≤ 0,12	≤ 0,5	≥ 16	32	≤ 2	8	4	≥ 3/20
**IHIT32403**	≥ 32	4	16	≤ 4	≤ 0,25	≤ 1	≤ 1	≤ 1	≤ 2	2	≤ 1	≤ 0,12	≤ 0,5	≥ 16	≤ 16	16	8	8	≥ 3/20
**IHIT32401**	≥ 32	4	≥ 128	≤ 4	≤ 0,25	≤ 1	≤ 1	≤ 1	≤ 2	≤ 1	≤ 1	≤ 0,12	≤ 0,5	≥ 16	≤ 16	≤ 2	4	4	≥ 3/20
**IHIT32402**	≥ 32	4	≥ 128	≤ 4	≤ 0,25	≤ 1	≤ 1	≤ 1	≤ 2	≤ 1	≤ 1	≤ 0,12	≤ 0,5	≥ 16	≤ 16	≤ 2	8	8	≥ 3/20

AMP = ampicillin, AMC = amoxicillin/clavulanic acid, PIP = piperacillin, LEX = cephalexin, CPD = cefpodoxim, CFT = ceftiofur, CEP = cefpirom, IPM = imipenem, AMK = amikacin, GEN = gentamicin, TOB = tobramycin, ENR = enrofloxacin, MAR = marbofloxacin, TET = tetracycline, NIT = nitrofurantoin, CHL = chloramphenicol, PMB = polymyxin B, CST = colistin, SXT = trimethoprim/sulfamethoxazole.

*MICs for colistin were determined using the Micronaut system (Merlin Diagnostika GmbH, Germany); all other antibiotics were tested by using the VITEK2 compact system (bioMérieux, Nürtingen, Germany).

Supplementary Table 3: Virulence-associated genes (90% coverage, ≥ 90% identity) in *mcr-2* positive *E. coli* isolates and predicted pathotype

Strain	Virulence associated genes and categories*				Predicted pathotype
	Adhesion	Toxin, hemolysin	Iron acquisition	Colicins, serum resistance, secretion and effector proteins	
IHIT31008	*ecpA-D, faeCDEFH, fdeC, hra, matB*	*astA, hlyA-D*	*entBCDEFS, fepABCD, fes, fyuA, iha, irp2, ybt*	*capU, cia, espL1, espX1, espX4, espX5, ompA, traT*	none
IHIT32395	*ecpA-D, faeCDEFH, fdeC, hra, matB, paa*	*astA, eltA, estb, hlyA-D*	*entBCDEFS, fepABCD, fes, fyuA, iha, irp1, irp2, ybt*	*capU, cia, espL1, espX1, espX4, espX5, ompA, traT*	ETEC
IHIT32396	*ecpA-D, faeCDEFH, hra, matB*	*astA, eltA, estb, hlyA-D*	*entBCEFS, fepABCD, fes, fyuA, iha, irp1, irp2, ybt*	*capU, cia, espL1, espX1, espX5, traT*	ETEC
IHIT32302	*ecpA-D, fedA, fedF, fimBCDEFHI* (*fimH24*), *matB*	*astA, eltA, estb, hlyA-D*	*chuASUWY, entBC, fepAD, fes, fyuA, iha, irp1, irp2, ybt*	*cba, cea, cib, cma, espX2, iss, ompA, ompT, traT*	ETEC
IHIT32399	*ecpA-D, fedA, fedF, fimA-I* (*fimH24*), *matB*	*astA, eltA, estb, hlyA-D*	*entBCDEFS, fepABDCG, fes,iha*	*cba, cea, cib, cma, espL1, espL4, espX1, espX4, espX5, espY1, iss, ompA, ompT, traT*	ETEC
IHIT32303	*ecpA-D, fed, fimBCDEFHI* (*fimH445*), *matB*	*astA, estb*	*chuASUWY, entBC, fepAD, fes, iha, irp1, irp2, fyuA, ybt*	*air, cea, eilA, espX2, traT*	ETEC
IHIT32397	*ecpA-D, fimB-I* (*fimH45*), *hra, matB*	*astA, estb*	*entBCDEFS, fepABCD, fes*	*cib, espL1, espL4, espX5, traJ*	ETEC-like**
IHIT32305	*ecpA-D, faeCDEFHJ, matB*	*astA, eltA, estb, hlyA-D*	*entBCEFS, fepABCD, fes, iha*	*cib, espL1, espX1, espX5, traJ*	ETEC
IHIT32304	*ecpA-D, fimB-I* (*fimH45*), *hra, matB*	*astA, estb*	*entBCDEF, fepABC, fes*	*cib, espL1, espL4, espX5, traJ*	ETEC-like**
IHIT32403	*aidA, aah, cfa, csgB, eae, ecpB-D, efa1, fed, fimB-I* (*fimH24*), *hra, lpf, paa, perABC, tsh*	*astA*	*entBCEFS, fepABCD, fes, iha*	*cma, cif, escCDEFGIJL, escN-V, espL2, espM1, espW, espX5, iss, katP, lifA, nleA-F, nleH, ompA, ompT, malX, sep, tccP, tir*	atypical EPEC
IHIT32401	*aah, fim* (*fimH25-like*), *hra*	*astA, estb*	*entBCDEFS, fepABCD, fes*	*cib, espL1, espX1, espX5, kpsD, kpsE, kpsM, kpsMII, ompA, traJ, traT*	ETEC-like**
IHIT32402	*aah, ecpA-D, fdeC, fimB-I* (*fimH27*), *matB*	*astA, estb*	*entBCDEFS, fepABCD, fes*	*espL1, espX1, espX5, iss, kpsD, kpsE, ompA, ompT*	ETEC-like**

The data result from a search of genome sequences by ResFinder 4.1 (https://cge.food.dtu.dk/services/ResFinder/) and BacWGSTdb (http://bacdb.cn/BacWGSTdb/Tools.php).

***Fimbrial/adhesin genes:** AIDA-I (adhesin involved in diffuse adherence), *aah* (AIDA-associated heptosyltransferase), *cfa* (CFA/I fimbriae), *csg* (curli fimbriae), *eae* (intimin), *ecp* (*E. coli* common pilus), *efa1* (EHEC factor for adherence), *fae* (F4 fimbriae), *fdeC* (factor adherence *E. coli*), *fed* (F18 fimbriae), *fim* (type I fimbriae), *hra* (heat-resistant hemagglutinin), *lpfA* (long polar fimbriae), *paa* (porcine attaching-effacing associated protein), *perABC* (regulatory operon bundle-forming pili), *tsh* (temperature-sensitive hemagglutinin).

Toxin/hemolysin genes: *astA* (enteroaggregative *E. coli* heat-stable enterotoxin 1), *eltA* (*E. coli* heat-labile enterotoxin A), *estb* (*E. coli* heat-labile enterotoxin B), *hly* (hemolysin)

Iron acquisition: *chuA* (hemin receptor), *ent* (enterobactin), *fep* (ferrienterobactin), *fes* (ferric enterobactin esterase), *fyuA* (yersiniabactin receptor), *iha* (enterobactin receptor), *irp* (iron repressible proteins), *ybt* (yersiniabactin).

Colicins, serum resistance, secretion and effector proteins: *capU* (hexosyltransferase homologue), *cba* (colicin-B), *cea* (colicin-E1), *cia* (colicin-Ia), *cib* (colicin-Ib), *cif* (cycling inhibiting factor, cyclomodulin), *cma* (colicin-M), *esp* (*E. coli* secreted proteins), *iss* (increased serum survival protein), *katP* (EHEC catalase peroxidase), kps (capsule biosynthesis), *lifA* (lymphostatin), *nle* (non-LEE encoded effector proteins), *ompA/T* (outer membrane proteins), *malX* (PAI marker, phosphotransferase enzyme), *sep* (secreted *E. coli* proteins), *tccP* (Tir-cytoskeleton coupling protein), *tir* (translocated intimin receptor), *traJ* (regulator transfer proteins), *traT* (complement resistance protein).

**The term "ETEC-like" was used for isolates that encoded for heat-labile and/or heat-stable toxins but lacked genes of ETEC-typical adhesive fimbriae.

Supplementary Table 4: Plasmid incompatibility groups and estimated plasmid sizes of *mcr-2* positive *E. coli* from pigs

Strain	Month / year of isolation	Country	Farm-Pig-Isolate	MLST	Plasmid incompatibility groups*	Estimated plasmid sizes (kb, S1-nuclease restriction)
IHIT31008	02/2015	BE	I-1-1	ST100	IncFII, IncI2, IncX1-like, IncX4	< 20, 47, 65, 150
IHIT32395	02/2015	BE	I-1-2	ST100	IncFIB-like, IncFII, IncFII(pSE11)-like, IncI2, IncX4	< 20, 47, 65, 170
IHIT32396	02/2015	BE	I-2-1	ST100	IncFIB-like, IncFII, IncFII(pSE11)-like, IncI2, IncX4	< 20, 47, 65, 170
IHIT32302	06/2015	BE	II-1-1	ST5786	IncFIA, IncFIB-like, IncHI1A-like, IncHI1B, IncI1-I(alpha), IncX4, p0111-like	< 20, 35, 110, 125, 220
IHIT32399	06/2015	BE	II-2-1	ST5786	IncFIA, IncFIB-like, IncHI1A-like, IncHI1B, IncI1-I(alpha), IncX4, p0111-like	< 20, 35, 110, 125, 220
IHIT32303	06/2015	BE	II-3-1	ST3057	IncFII, IncHI2, IncHI2A, IncX4, IncY-like	35, 95, 125, 270
IHIT32397	05/2015	BE	III-1-1	ST10	IncFII, IncI1-I(alpha), IncQ1-like, IncX4	< 20, 35, 110, 125
IHIT32305	05/2015	BE	III-2-1	ST100	IncB/O/K/Z-like, IncFIB-like, IncFIC-like, IncFII-like, IncI-like, IncX4, p0111-like	< 20, 35, 60, 85, 95, 170
IHIT32304	05/2015	BE	III-3-1	ST10	IncFII, IncI1-I(alpha), IncQ1-like, IncX4	< 20, 35, 110, 125
IHIT32403	10/2013	ES	IV-1-1	ST29	IncFIA-like, IncHI2, IncHI2A-like, IncI1-I, IncQ1-like, IncX4	35, 110, 140, 250
IHIT32401	08/2014	DE	V-1-1	ST93	IncFII, InHI2, IncHI2A-like, IncQ1-like, IncX4, IncY-like	< 20, 35, 95, 150, 250
IHIT32402	12/2014	DE	VI-1-1	ST11875	IncFII, InHI2, IncHI2A-like, IncQ1-like, IncX4, IncFIA, IncI1-I	35, 85, 95, 270

BE = Belgium, DE = Germany, ES = Spain; *threshold for minimum identity: 98%, minimum coverage range: 60%

Supplementary Table 5: Characteristics of plasmids that share a common backbone to the novel 47-kb MCR-2 plasmid of porcine *E. coli* IHIT31008

Name[a]	GenBank	Size	Bacterial host, serovar and sequence type (ST), phylogenetic group	Host/Source	Country	Year	Plasmid Inc-type[b]	Query cover plasmid/ identity	Identity entire plasmid
pCFSA664-2	CP033354.2	41,696	*S.* Enteritidis, ST17	Food	China	2015	ND	88.0%/100%	88.7%
pAUSMDU00010527	CP045957.1	41,464	*S.* Enteritidis, ST3304	Human	Australia	2017	ND	87.0%/99.91%	87.6%
pRHB15-C18_3	CP057780.1	41,696	*E. coli*, ST8185, B1	Pooled sheep faecal sample (floor)	UK	2017	ND	88.0%/99.99%	88.7%
pRHB23-C01_5	CP057569.1	41,486	*E. fergusonii*	Pooled sheep faecal sample (floor)	UK	2017	ND	88.0%/99.90%	88.1%
pYDC107_41	CP025711.1	41,544	*E. coli* O102:H6, ST964, D	Human drainage	USA	2008	ND	84.0%/97.63%	84.6%
Res13-Lact-PEA12-26 plasmid_unnamed_novel_0	CP062878.1	46,158	*E. coli* O157:H16, ST5502, A	Pig feces	Canada	2017	ND	85.0%/99.67%	77.6%
pTHO-015-2	AP022551.1	46,459	*E. coli* O25:H4, ST131, B2	Human urine	Japan	2018	ND	85.0%/96.95%	79.9%

[a] By using ResFinder 4.1 (https://cge.food.dtu.dk/services/ResFinder/), antimicrobial resistance genes were only detected in plasmid pTHO-015-2 ($bla_{\text{CTX-M-14}}$).

[b] ND indicates that the plasmid incompatibility typing, using PlasmidFinder (https://cge.food.dtu.dk/services/PlasmidFinder/), gave no result.

Supplementary Table 6: Occurrence of *mcr-2* and other *mcr* genes in samples from pigs and other animals, from animal meat, humans and the environment according to 104 publications. Only studies that included a screening for the *mcr-2* gene were considered.

Country[a]	Year of isolation	Source (no.) of samples[b]	Bacterial species[c], no. of isolates (no. of ColR isolates) [d]	*mcr-1*	*mcr-2*	*mcr-3 – mcr-10* no. of pos. isolates[e]	REF
				% (no. of positive isolates per no. of tested isolates)			
**EUROPE**							
BEL	2011-2012	Passive surveillance diarrhoea<br>• **P (53)**<br>• CA (52)	Ec: 105 ColR	12.4% (13/105)<br>P: 13.2% (7/53)<br>CA: 11.5% (6/52)	11.4% (12/105)<br>P: 20.8% (11/53)<br>CA: 1.9% (1/52)	n.t.	[1]
BEL	2012-2015	• Food chain – POUL, **pork**, others (National surveillance program)	Sal: 1,415 (105 ColR)	1.9% 2/105 [pork carcass, 2012]	0.95% (1/105)<br>[pork carcass, S. Derby, ST40, pKP37-BE-like IncX4, 2012]	n.t.	[2]
BEL	2012-2016	• **P – healthy, fattening farm (4)**<br>• CA – healthy veal calves (32)<br>• POUL - healthy (1)<br>• CA meat (3)	Ec: 40 ColR	77.5% (31/40)<br>[1 *mcr-1* & *mcr-3* & *mcr-5*]	2,5% (1/40)<br>[P, 2016, pKP37-like IncX4]	*mcr-3* (1)<br>*mcr-4* (8)<br>*mcr-5* (1)<br>[1 *mcr-1* & *mcr-3* & *mcr-5*]<br>*mcr-6 – mcr-10* n.t.	[3]
CHE	2008-2018	• H - clinical	Ent: 97 (including 4 reference samples)	3.2% (3/93)	0% (0/93)	n.t.	[4]
CHE	2016	Healthy animals at slaughter, caecal<br>• **P (325)**<br>• CA (241)<br>• POUL (100)	Ent (Hal, Ec, Ecl, Kp)<br>P: 4% ColR (13/325)<br>CA: 3.3% (8/241)<br>POUL: 0% (0/100)	0% (0/21)	0% (0/21)	n.t.	[5]
CHE	2016	• H – UTI, urine	Ent: 2,049 (6 ColR)	0.05% (1/2,049)	0% (0/6)	n.t.	[6]
CHE	2016	• H – healthy, stool (1,091)<br>• H – primary care patients, stool (53)	62 Ent selective plates (4 mg/L Col) (18 ColR - Hal: 9, Ec: 3, Ecl: 4, Kp: 1, Rorn: 1)	0% (0/62)	0% (0/62)	n.t.	[7]
CZE	2018-2019	• H – hospitalised, rectal swabs and stool samples	enriched cultures of 1,922 samples (qPCR)	0.21% (4/1,922)	0% (0/1,922)	*mcr-3* to *mcr-8* (0)<br>*mcr-9, -10* n.t.	[8]
DEU	2011-2012	• **P – healthy, 58 fattening farms**; boot swabs, pooled fecal samples	various GN species: 436	9.9% samples;<br>25.9% farms	0% (0/436)	n.t.	[9]
DEU	2011-2018	• Healthy animals (**P**, POUL, CA, Pet), food (POUL meat, **pork**)	Sal: 407 ColR	45% (183/407)<br>[8 *mcr-1* & *mcr-9*]	0% (0/407)	*mcr-3* (0)<br>*mcr-4* (53)<br>*mcr-5* (18)<br>*mcr-6, -7, -8* (0)<br>*mcr-9* (8)<br>[8 *mcr-1* & *mcr-9*]	[10]

Country[a]	Year of isolation	Source (no.) of samples[b]	Bacterial species[c], no. of isolates (no. of ColR isolates) [d]	*mcr-1*	*mcr-2*	*mcr-3 – mcr-10* no. of pos. isolates[e]	REF
				% (no. of positive isolates per no. of tested isolates)			
						mcr-10 n.t.	
DEU	2016-2017	• Municipal water - metagenome analysis	14 samples	71.4% of samples (1/14)	0% of samples (0/14)	*mcr-3, -4, -5, -7* ubiquitous in all samples<br>*mcr-2, -6, -8, -9* (0)<br>*mcr-10* n.t.	[11]
DNK	2009-2017	• H - clinical	Sal: ca. 2,500 genomes	0.04% (1/2500)<br>[1 *mcr-1* & *mcr-3*]	0% (0/2,500)	*mcr-3* (10)<br>[1 *mcr-1* & *mcr-3*]<br>*mcr-4 – mcr-10* n.t.	[12]
DNK	2014-2017	• H – blood stream infection	Ec ESBL/AmpC: 872 genomes<br>CPOs: 317 genomes	0.08% (1/1,183)	0% (0/1,183)	*mcr-3* (1)<br>*mcr-4 – mcr-10* n.t.	[13]
ESP	2005-2014	• **P – PWD diagnostic samples**<br>• White stork – fecal droppings	P – Ec: 70 (10/year)<br>White stork – Ec: 20	P: 20.0% (14/70)<br>White stork: 25.0% (5/20)	0% (0/90)	*mcr-4* (1)<br>*mcr-3, -5* (0)<br>*mcr-6 – mcr-10* n.t.	[14]
ESP	2006-2016	• **P – enteric colibacillosis**	Ec: 35 *mcr*-positive (35/499 investigated isolates)	3.8% (19/499)	0% (0/499)	*mcr-3* (0)<br>*mcr-4* (18)<br>*mcr-5* (2)<br>*mcr-6 – mcr-10* n.t.	[15]
ESP	2006-2017	• **P – PWD**	Ec: 186 ETEC & STEC (126 ColR)	29.4% (37/126)<br>[1 *mcr-1* & *mcr-4*,<br>1 *mcr-1* & *mcr-5*]	0% (0/126)	*mcr-3* (0)<br>*mcr-4* (102/126)<br>[1 *mcr-1* & *mcr-4*]<br>*mcr-5* (5/126)<br>[1 *mcr-1* & *mcr-5*]<br>*mcr-6 –mcr-10* n.t.	[16]
ESP	2001	• **P –** healthy, nasal turbinate	*Moraxella pluranimalium* sp. nov.: 1 isolate	-	positive	*mcr-3 – mcr-10* n.t.	[17, 18]
ESP	2015	• CA – healthy, < 1 year, slaughterhouse, caecal content (636 animals, 318 farms)	GN: 152 potential ESBL/AmpC (6 ColR)	100% (6/6)<br>[1 *mcr-1* & *mcr-3*]	0% (0/6)	*mcr-3* (1)<br>[1 *mcr-1* & *mcr-3*]<br>*mcr-4 – mcr-10* n.t.	[19]
ESP	2018	• **P – slaughterhouses, caecal content (272)**	GN: 486 (249 Ec)	54,1% (263/486)	0.2% (1/486)<br>[Ec, O83:H42, ST648, 2018]	n.t.	[20]
EUR	2002-2014	Healthy animals at slaughter<br>• CA (3,101)<br>• **P (4,563)**<br>• CH (4,316)	Ec: 10,206, Sal: 1,774<br>CA: 36 ColR<br>P: 85 ColR<br>CH: 119 ColR	Ec: 0.7%, Sal: 0.1%<br>CA: 0% (0/36)<br>P: 29.4% (25/85)<br>CH: 37.8% (45/119)	0% (0/240 ColR)	n.t.	[21]
FRA	2016	• H – patients (653), rectal swabs	GN: 9 with acquired ColR	0% (0/9)	0% (0/9)	n.t.	[22]
FRA	2017	• CA – veal calves at slaughter (170)	Ec: 268 (27 ColR)	12.7% (34/268)<br>[3 *mcr-1* & *mcr-3*]	0% (0/268)	*mcr-3* (10)<br>[3 *mcr-1* & *mcr-3*]<br>*mcr-4 – mcr-10* n.t.	[23]

Country[a]	Year of isolation	Source (no.) of samples[b]	Bacterial species[c], no. of isolates (no. of ColR isolates) [d]	*mcr-1*	*mcr-2*	*mcr-3 – mcr-10*	REF
				% (no. of positive isolates per no. of tested isolates)		no. of pos. isolates[e]	
FRA	2019-2020	Healthy pets in shelters, feces<br>• dogs (52)<br>• cats (105)	GP: 154<br>GN: 64	6.4% (14/218)	0% (0/218)	*mcr-3, -4, -5, -8* (0)<br>*mcr-6, -7, -9, -10* n.t.	[24]
FRA	2020	**P – healthy, 2-8 months, fecal samples (80)**	Extracted DNA samples (80)	1.3% (1/80) [*E. coli*]<br>[1 *mcr-1* & *mcr-3*]	0 (0/80)	*mcr-3 (1)*<br>[1 *mcr-1* & *mcr-3*]<br>*mcr-4, -5, -8* (0)<br>*mcr-6, -7, -9, -10* n.t.	[25]
GBR	2014-2015	• **P – healthy, at abattoirs, caecal content (57 farms)**	Ec, *Klebsiella*, Sal, *Moraxella*: 657	0.15% (1/657) [*M. porci*]	0.15% (1/657)<br>[*M. pluranimalium*-like]	*eptA* genes: 0.9% (6/657)<br>[*M. osloensis*]<br>*mcr-3 – mcr-10* n.t.	[26]
GBR	2014-2017	Public health surveillance<br>• H, food, animal environment	Sal: 33,205 genomes	0.1% (32/33,205)	0% (0/33,205)	*mcr-3* (19)<br>*mcr-4* (0)<br>*mcr-5* (1)<br>*mcr-6 – mcr-8* (0)<br>*mcr-9, -10* n.t.	[27]
GBR	2019	• CA – calves, experimental study	Microbiota after florfenicol treatment	no	yes	n.t.	[28]
ITA	2000-2020	Feces, organs<br>• Arthropoda: housefly, crayfish (13); birds (51); mammals (63); reptiles (98)<br>• Environment/feed (11)	Sal: 236 (42 ColR)	0.84% (2/236)<br>[1 *mcr-1* & *mcr-4*]	2.96% (7/236)<br>[1 *mcr-2* & *mcr-4*]<br>[1 isolate each of donkey, sheep, housefly; 4 of reptiles]	*mcr-4* (4)<br>[1 *mcr-1* & *mcr-4*,<br>1 *mcr-2* & *mcr-4*]<br>*mcr-6* (1)<br>*mcr-8* (2)<br>*mcr-3, -5, -7, -9* (0)<br>*mcr-10* n.t.	[29]
ITA	2013-2017	• H – patients	Kp: 369 CPR (127 Col-non-S)	0% (0/127)	0% (0/127)	n.t.	[30]
ITA	2014/2015	• Fattening turkey<br>• Broiler chickens<br>• **P – fattening farm**<br>• Bovines < 12 months	Turkey – iEc: 170 (39 ColR),<br>ESBL/AmpC Ec: 224 (58 ColR),<br>Sal: 146 (12 ColR)<br>Broilers – iEc: 170 (9 ColR),<br>ESBL/AmpC Ec: 244 (13 ColR)<br>Sal: 90 (0 ColR)<br>Pig – iEc: 168 (1 ColR),<br>ESBL/AmpC Ec: 214 (14 ColR)<br>Bovines – iEc: 170 (8 ColR),<br>ESBL/AmpC Ec: 179 (7 ColR)	Turkey: iEc: 97.4% (38/39),<br>ESBL/AmpC Ec: 100% (58/58),<br>Sal: 25.0% (3/12)<br>Broilers: iEc: 88.9% (8/9),<br>ESBL/AmpC Ec: 84.6% (11/13)<br>Sal: n.t.<br>Pig: iEc: 100% (1/1),<br>ESBL/AmpC Ec: 92.9% (13/14)<br>Bovines: iEc: 62.5% (5/8),<br>ESBL/AmpC Ec: 57.1% (4/7)	0%	*mcr-3* (4, bovine ESBL/AmpC Ec)<br>*mcr-4* (2, ESBL/ AmpC Ec pig & iEC bovine)<br>*mcr-5* (0)<br>*mcr-6 – mcr-10* n.t.	[31]
ITA	2015-2016	• **P – PWD**	Ec: 51	72.5% (37/51)	0% (0/14 *mcr-1* neg.)	n.t.	[32]
ITA, ESP, BEL	2015-2016	• **P – PWD**	Ec: 125 (50 ColR)<br>ITA: 34, Spain: 43, BEL: 48	25.6% (32/125)	2.4% (3/125)<br>(BEL: P 2, CA 1; see Ref. [1])	*mcr-4* (11)<br>*mcr-3* (0)	[33]

Country[a]	Year of isolation	Source (no.) of samples[b]	Bacterial species[c], no. of isolates (no. of ColR isolates) [d]	*mcr-1* % (no. of positive isolates per no. of tested isolates)	*mcr-2*	*mcr-3 – mcr-10* no. of pos. isolates[e]	REF
						mcr-5 – mcr-10 n.t.	
ITA	2016-2017	• H – clinical	Ent: 19,053 (12,441 Ec) (90 ColR)	28.9% (26/90), all Ec	0% (0/90)	*mcr-3, -4, -5* (0)<br>*mcr-6 – mcr-10* n.t.	[34]
ITA	2016-2017	POUL<br>• Broiler – cloacal swabs (13)<br>• Slaughterhouse – product samples (72): environmental, skin, liver, meat	Sal: 85 (6 ESBL, 3 ColR)	3.5% (3/85)	0% (0/85)	*mcr-3 – mcr-5* (0)<br>*mcr-6 – mcr-10* n.t.	[35]
ITA	2018-2019	• Wild boar – hunted	Ec: 168 (47 ColR)	29.2% (49/168)<br>[23 *mcr-1* & *mcr-2*]	29.2% (49/168)<br>[23 *mcr-1* & *mcr-2*]	n.t.	[36]
NLD	2014-2015	• H – patients attending hospital (576)	621 fecal samples	0.35% (2/576 patients)	0% (0/576)	n.t.	[37]
NLD, DEU, DNK, BEL	2015	• CH – retail meat (214)	Extracted DNA samples (214)<br>NLD: 67<br>DEU: 44<br>DNK: 9<br>NLD/DEU: 80<br>NLD/DEU/BEL: 12<br>Unknown: 2	24.8% (53/214)<br>NLD: 30.3% (21/67)<br>DEU: 43.2% (19/44)<br>DNK: 11.1% (1/9)<br>NLD/DEU: 13.8% (11/80)<br>NLD/DEU/BEL: 0% (0/12)<br>Unknown: 50.0% (1/2)	0% (0/214)	n.t.	[38]
POL	2011-2016	Fecal samples<br>• Turkeys, broilers, layers (74), **P (1)**, and CA (1)	Ec: 5,878 (128 ColR)	62.5% (80/128 ColR)	0% (0/128)	*mcr-3, -4, -5* (0)<br>*mcr-6 – mcr-10* n.t.	[39]
ROU	2014-2017<br>2011-2012<br>2011-2012	• H – clinical<br>• H – poultry abattoir workers<br>• Broilers – fecal	H – clinical: Ent: 543 ColR and/or CPR<br>H – abattoir workers: 15 Ec, 3GCR<br>Broilers: 92 Ec, 3GCR	0% (0/543)<br>0% (0/15)<br>11.9% (11/92)	0% (0/543)<br>0% (0/15)<br>0% (0/92)	n.t.	[40]
**Non-European countries**							
BGD	not specified	• H (100), CA (50), goat (100), POUL (250)<br>• POUL – farm environment (150)<br>• Street food (160)	Ec: 358	0.73% (3/410)<br>[3 POUL isolates]	0.49% (2/410)<br>[2 street food isolates]	n.t.	[41, 42]
BGD	2017-2018	• H – UTI, urine (142)	Ent: 123	9.8% (12/123)	1.6% (2/123)<br>[Ec & Kp]	*mcr-3, -4, -5* (0)<br>*mcr-6 – mcr-10* n.t.	[43]
BGD	2017-2018	• POUL – healthy, feces (104)	Ec: 104 ESBL (98 ColR)	13.5% (14/104)	0% (0/104)	*mcr-3, -4, -5* (0)<br>*mcr-6 – mcr-10* n.t.	[44]
BGD	2017-2018	• POUL – dropping samples (100; 20 farms)	Ent: 149 (92 ColR)	28.9% (43/149)<br>[2 *mcr-1* & *mcr-2*]	3.4% (5/149)<br>[2 *mcr-1* & *mcr-2*]	*mcr-3, -4, -5* (0)<br>*mcr-6 – mcr-10* n.t.	[45]

**Country**[a]	**Year of isolation**	**Source (no.) of samples**[b]	**Bacterial species**[c]**, no. of isolates** (no. of ColR isolates) [d]	***mcr-1***	***mcr-2***	***mcr-3 – mcr-10***<br>no. of pos. isolates[e]	**REF**
				% (no. of positive isolates per no. of tested isolates)			
BGD	2018	• POUL (20 broiler farms, 3 sampling times, 60 pooled samples)	Ec: 1,200	25.4% (305/1,200)	0% (0/1,200)	*mcr-3, -4, -5* (0)<br>*mcr-6 – mcr-10* n.t.	[46]
BOL	2016	• H – children, healthy, feces	GN: 337 (cultivated on MacConkey with 2 mg/L Colistin)	38.3% (129/337)	0% (0/337)	n.t.	[47]
BRA	2013-2016	• POUL – meat cuts (33 cuts, 24 markets)	Sal: 60 (7 ColR)	3.3% (2/60)	0% (0/60)	*mcr-3, -4* (0)<br>*mcr-5 – mcr-10* n.t.	[48]
BRA	2015-2016	• POUL – healthy, trachea/cloaca (107) & APEC (2)	Ec: 109	57.9% (62/109)	0% (0/109)	*mcr-3, -4* (0)<br>*mcr-5* (3)<br>*mcr-6* - mcr-*9* (0)<br>*mcr-10* n.t.	[49]
KHM	2017	• **P – healthy, feces (91 farms)**	Ec: 261 (52 ColR)	80.8% (42/52)<br>[11 *mcr-1* & *mcr-3*]	0% (0/52)	*mcr-3* (20)<br>[11 *mcr-1* & *mcr-3*]<br>*mcr-4, -5* (0)<br>*mcr-6 – mcr-10* n.t.	[50]
CAN	2011-2012	• CH – healthy, feces (12)	Ec potential ESBL/AmpC: 108 (0 ColR)	0% (0/108)	0% (0/108)	n.t.	[51]
CAN	2017	• **P – healthy; fecal microbiota** (5 sampling dates over growing period)	Extracted DNA samples (fecal samples 33-62 pigs per sampling date)	positive at 5 sampling dates	positive at 3 sampling dates	n.t.	[52]
CHN	2004-2012	• **P,** CA, CH, ducks – clinical	Total - Ec: 624<br>P - Ec: 113<br>CH - Ec: 404<br>Ducks - Ec: 44<br>CA Ec: 63	Total: 2.7% (17/624)<br>P: 0.9% (1/113)<br>CH: 3.2% (13/404)<br>Ducks: 6.8% (3/44)<br>CA: 0% (0/63)	0% (0/624)	*mcr-3* (0)<br>*mcr-4 – mcr-10* n.t.	[53]
CHN	2008-2014	• CH – cloacal swabs	Ec: 821	44 among ColR isolates (total no. of ColR unclear)	0%	*mcr-3, -4, -5* (0)<br>*mcr-6 – mcr-10* n.t.	[54]
CHN	H: 2011-2014<br>C: 2013	• H – clinical (2,353 Ent)<br>• CH – slaughterhouse	H: 964 Kp (6 ColR), 1,389 Ec (23 ColR)<br>CH: 47 Kp (11 ColR), 121 Ec (10 ColR)	H: 0.4% (4/964) Kp, 1.7% (23/1,389) Ec<br>CH: 0% Kp, 8.3% (10/121) Ec	0% (0/13 *mcr-1* neg. & ColR)	n.t.	[55]
CHN	2013	• Flies on a university campus (297)	Extracted DNA samples: 297<br>GN: 189	DNA: 36.7% (109/297)<br>GN: 4.8% (9/189)	DNA: 1.4% (4/297)<br>GN: 0% (0/189)	DNA: *mcr-3* (33)<br>GN: *mcr-3* (0)<br>*mcr-4 – mcr-10* n.t.	[56]
CHN	2014, 2016	• **P – healthy, feces (1,552)**<br>• POUL – healthy, nasal, oropharyngeal, anal/cloacal swabs (1,836)	Extracted DNA samples:<br>P: 1,454<br>CH: 1,498<br>Geese: 109<br>Ducks: 130<br>Pigeons: 99	P: 79.2% (1,152/1,454)<br>CH: 31.8% (476/1,498)<br>Geese: 71.7% (78/109)<br>Ducks: 5.5% (7/130)<br>Pigeons: 13.1% (13/99)	P: 56.3% (819/1,454)<br>CH: 5.5% (82/1,498)<br>Geese: 5.5% (6/109)<br>Ducks: 2.3% (3/130)<br>Pigeons: 0% (0/99)	*mcr-3:*<br>P: 18.7%<br>CH: 5.2%<br>Geese: 11.9%<br>Ducks: 13.8%<br>Pigeons: 5.1%	[57]

Country[a]	Year of isolation	Source (no.) of samples[b]	Bacterial species[c], no. of isolates (no. of ColR isolates) [d]	*mcr-1* % (no. of positive isolates per no. of tested isolates)	*mcr-2*	*mcr-3 – mcr-10* no. of pos. isolates[e]	REF
						mcr-4 – mcr-10 n.t.	
CHN	2015-2016	• **P**, CH, CA – healthy, intensive feeding farms, fecal swabs (2,199)	P – Ec: 811 (440 Col-R)<br>CH – Ec: 1,232 (443 Col-R)<br>CA – Ec: 156 (42 Col-R)	P: 68.9% (303/440)<br>[88 *mcr-1* & *mcr-2*]<br>CH: 87.6% (388/443)<br>[32 *mcr-1* & *mcr-2*]<br>CA: 71.4% (30/42)<br>[4 *mcr-1* & *mcr-2*]	PI: 46.8% (206/440)<br>[88 *mcr-1* & *mcr-2*]<br>CH: 14.9% (66/443)<br>[32 *mcr-1* & *mcr-2*]<br>CA: 19.1% (8/42)<br>[4 *mcr-1* & *mcr-2*]	n.t.	[58]
CHN	2016	• H – women attending hospital, vaginal swabs (134)	Extracted DNA samples	0.7% (1/134)<br>[*mcr-1* & *mcr-5*]	1.5% (2/134)	*mcr-3* (2)<br>*mcr-4* (17)<br>*mcr-5* (1)<br>[*mcr-1* & *mcr-5*]<br>*mcr-6 – mcr-10* n.t.	[59]
CHN	2016	• Farming soil samples (96)	Ec ESBL: 42 (10 ColR)<br>Kp ESBL: 11 (2 ColR)	50 % (6/12 ColR)	0% (0/12 ColR)	*mcr-3, -4* (0)<br>*mcr-5 – mcr-10* n.t.	[60]
CHN	2019	• cats and dogs – healthy and clinical, fecal samples (1550)	Kp: 1190 (ColR)	12.5% (149/1190)<br>[4 *mcr-1* & *mcr-3*,<br>3 *mcr-1* & *mcr-5*]	0.9% (11/1190)	*mcr-3* (15) [4 *mcr-1* & *mcr-3*]<br>*mcr-4* (6)<br>*mcr-5* (16) [3 *mcr-1* & *mcr-5*]<br>*mcr-7, -8* (0)<br>*mcr-9* (5)<br>*mcr-10* (4)<br>*mcr-6* n.t.	[61]
EGY	2017-2018	• Resident wild birds (80)<br>• Migratory waterfowls (60)<br>• Surface water (20)<br>• H – farmer, stool (50)	RB – Ec: 33, Kp: 22, Kox: 4, Pa: 8<br>MB – Ec: 29, Kp: 9, Kox: 6, Pa: 11<br>SW – Ec: 7, Kp: 7, Kox: 1, Pa: 3<br>H – Ec: 10, Kp: 15, Kox: 2, Pa: 4	RB: 10.4% (7/67)<br>MB: 20.6% (11/55)<br>[2 *mcr-1* & *mcr-2*]<br>SW: 16.6%<br>H: 9.6%<br>[1 *mcr-1* & *mcr-2*]	RB: 1.4% (1/67, Pa)<br>MB: 3.6% (2/55, Ec, Kp)<br>[2 *mcr-1* & *mcr-2*]<br>SW: 11.1% (2/18, Ec, Kp)<br>H: 9.6% (3/31, Ec, Kp)<br>[1 *mcr-1* & *mcr-2*]	n.t.	[62]
EGY	2018-2020	• CA – milk samples (total number not given)<br>• clinical mastitis (70), subclinical mastitis (11), raw milk (36)	Ec: 42 (24 ColR)<br>Cb: 20 (0 ColR)<br>Kp: 18 (14 ColR)<br>Am: 17 (15 ColR)<br>Pa: 10 (8 ColR)<br>Ent: 10 (0 ColR)	Ec: 4.8% (2/42)<br>Kp: 22.2% (4/18)<br>Am: 37.5% (6/16)<br>Pa: 30% (3/10)	Ec: 19% (8/42)<br>Kp: 16.7% (3/18)<br>Am: 12.5% (2/16)<br>Pa: 10% (1/10)	*mcr-3* (16)<br>*mcr-4* (1)<br>*mcr-7* (1)<br>*mcr-5, -6, -8, -9* (0)<br>*mcr-10* n.t.	[63]
EGY	2018-2020	• CA – milk samples (570)	Ec: 90 (24 ColR)<br>Pm: 33 (4 ColR)	0% (0/10)	0% (0/10)	*mcr-3 – mcr-9* (0)	[64]

Country[a]	Year of isolation	Source (no.) of samples[b]	Bacterial species[c], no. of isolates (no. of ColR isolates) [d]	*mcr-1* % (no. of positive isolates per no. of tested isolates)	*mcr-2*	*mcr-3 – mcr-10* no. of pos. isolates[e]	REF
		• clinical mastitis (350), subclinical mastitis (95), raw milk (125)	Kp: 25 (14 ColR)<br>Am: 17 (14 ColR)<br>Ecl: 15 (0 ColR)<br>Cb: 4 (0 ColR)			*mcr-10* (1 Kp from raw milk)	
EGY	2019	• H – patients (324 samples)	Ec & Kp: 200 (24 ColR)	8.3% (2/24)	0% (0/24)	n.t.	[65]
EGY	not specified	• H - clinical in ICU	Ec & Kp: 100 (70 ColR)	0% (0/100)	0% (0/100)	n.t.	[66]
GHA	2018-2019	• H - clinical	Ec: 135 (2 CPOs)	0.7% (1/135)	1.5% (2/135)	*mcr-3 – mcr-8* (0)<br>*mcr-9, -10* n.t.	[67]
HKG	2016	• H – routine stool samples (672) from 616 individuals	Ent: 79 Col-non-S	17.7% (14/79)	0% (2/79)	n.t	[68]
IND	2013-2015	• H – clinical	Kp: 8 ColR	0% (0/8)	0% (0/8)	n.t.	[69]
IND	2017-2018	• H – consecutive samples ocular infections	GN: 60 (24 Col-R, including 15 intrinsically resistant isolates)	6.3% (1/16 ColR tested)	25.0% (4/16 ColR tested, Bcep, Pa)	n.t.	[70]
IND	2018-2019	• H – clinical, urine (109), pus (24), respiratory (42), blood (5), miscellaneous (20)	Ec, CPOs: 113 (3 ColR)<br>Kp, CPOs: 79 (22 ColR)<br>Ent, CPOs: 8 (2ColR)	0% (0/27 ColR tested)	0% (0/27 ColR tested)	n.t.	[71]
IRN	2008-2016	• Various animal species	Broiler: 183 APEC; CA: 94 STEC, 36 MPEC; ostrich: 35 septicemic Ec, 70 fecal Ec; sheep: 51 STEC, 31 commensal Ec; pigeon: 33 STEC; dog: 74 commensal Ec	0% (0/607)	0% (0/607)	n.t.	[72]
IRN	2017	• H – clinical	Ec: 351 (38 Col-non-S)<br>Kp: 119 (26 Col-non-S)	Ec: 1.7% (6/351)<br>Kp: 1.7% (2/119)	Ec: 0% (0/351)<br>Kp: 0% (0/119)	n.t.	[73]
IRN	2019	• CA (38)<br>• CH (47)<br>• Urban sewage (30)	CA – Ec: 18 (1 ColR)<br>CH – Ec: 30 (1 ColR)<br>Urban sewage – Ec: 17 (1 ColR)	33.3% (1/3 ColR)	0% (0/3 ColR)	*mcr-3, -4, -5, -6* (0)<br>*mcr-7 – mcr-10* n.t.	[74]
IRN	not specified	• POUL – fecal samples (156 samples, 23 farms)	Sal: 30	0% (0/30)	0% (0/30)	n.t.	[75]
IRQ	2016-2018	• clinical and environmental samples	Ab: 121 (92 ColR)	73.5% (89/121)	64.5% (78/121)	*mcr-3* (82)<br>*mcr-4 – mcr-10* n.t.	[76]
KOR	2007-2016	• **P – diarrhoeic weaned piglets (100 pig herds)**	Ec: 364	1.1% (4/364)<br>[3 *mcr-1* & *mcr-3*]	0% (0/364)	*mcr-3* (8) [3 *mcr-1* & *mcr-3*]<br>*mcr-4 – mcr-10* n.t.	[77]
JPN	2000-2014	national veterinary antimicrobial resistance monitoring<br>• CA – healthy (3,134)	Ec: 9,306 (732 ColR)	All animals: 0.42% (39/,9306);<br>5.3% (39/732 ColR)<br>CA: 0.16% (5/3134)	0% (0/732)	n.t.	[78]

Country[a]	Year of isolation	Source (no.) of samples[b]	Bacterial species[c], no. of isolates (no. of ColR isolates) [d]	*mcr-1*	*mcr-2*	*mcr-3 – mcr-10*	REF
		% (no. of positive isolates per no. of tested isolates)				no. of pos. isolates[e]	
		• **P – healthy (2,052)**<br>• Broilers – healthy (2,017)<br>• Layers – healthy (2,103)		P: 0.97% (20/2,052)<br>Broilers: 0.69% (14/2,017)<br>Layers: 0% (0/2,103)			
JPN	2008-2015	• **P – healthy/PWD (120)**<br>• H – clinical (514)	Ec: 676	P – healthy: 2.4% (1/42)<br>P – PWD: 30% (36/120)<br>[5 *mcr-1* & *mcr-5*]<br>H: 0% (0/514)	0% (0/676)	*mcr-3* (10)<br>*mcr-4* (0)<br>*mcr-5* (35)<br>[5 *mcr-1* & *mcr-5*]<br>*mcr-6 – mcr-10* n.t.	[79]
JPN	2015	• Retail meat (111) - CH (55), **pork (32)**, beef (24)	CH – Ec: 154 (9 ColR)<br>Pork – Ec: 55 (1 ColR)<br>Beef – Ec: 47 (0 ColR)	CH: 5.2% (8/154)<br>Pork: 1.8% (1/55)<br>Beef: 0% (0/47)	0% (0/9 ColR)	n.t.	[80]
MYS	not specified	• Raw food samples – CH meat (50), bean sprouts (50)	CH meat – Ec: 23<br>Bean sprouts – Ec: 6	CH meat: 52.2% (12/23)<br>Bean sprouts: 0 (0/6)	0% (0/29)	n.t.	[81]
PAK	not specified (study over 18 months)	• H – clinical (38,500)<br>• POUL meat, CH fecal; respiratory secretion (630)	H: 5,893 GN (17 ColR)<br>Animal: 630 GN (126 ColR)	H: 23.5% (4/17)<br>Animal: 82.5% (104/126)	H: 0% (0/17)<br>Animal: 1.6% (2/126, Pm)	*mcr-3, -4, -5* (0),<br>*mcr-6* to *mcr-10* n.t.	[82]
PAK	not specified	• H – clinical (6,879)	GN: 718 (57 ColR)	31.6% (18/57)<br>[Ec, Kp, Ab, Pa]	1.8% (1/57)<br>[Kp, NDM-1/CTX-M-1]	*mcr-3, -4, -5* (0)<br>*mcr-6 – mcr-10* n.t.	[83]
PAK	not specified	• H – clinical	Kp: 200	12.0% (24/200)<br>[14 *mcr-1* & *mcr-2*]	8.5% (17/200)	n.t.	[84]
SEN	2011	• CH – healthy, feces (50)	Ec potential ESBL/AmpC: 93 (2 ColR)	0% (0/2)	0% (0/2)	n.t.	[51]
SGP	2017	• H – diarrheal stool samples (201)	Ent: 23 SuperPolymyxin screening agar (19 ColR/Col-non-S)	63.2% (12/19)	0% (0/19)	n.t.	[85]
THA	2007-2013	• **P – healthy (1), clinical (2)**	Ec ESBL: 3 (3 ColR)	66.7% (2/3)	33.3% (1/3)	*mcr-3* (2)<br>*mcr-4* (0)<br>*mcr-5 – mcr-10* n.t.	[86]
THA	2007-2018	• **P – healthy, fecal (354); clinical, fecal (100)**	Ec: 454 (217 ColR)	10.4% (47/454)<br>[32 *mcr-1* & *mcr-3*]	1.1% (5/454)<br>[5 *mcr-2* & *mcr-3*]	*mcr-3* (204)<br>[32 *mcr-1* & *mcr-3*, 5 *mcr-2* & *mcr-3]*<br>*mcr-4* (0)<br>*mcr-5 – mcr-10* n.t.	[87]
THA	2016-2019	• H – clinical	Ent: 6,996 MDR (4,516 ColR, thereof 4,235 CPE)	0.3% (13/4,235)<br>1.03% E<br>0.12% Kp<br>[1 Ec *mcr-1* & *mcr-3*]	0% (0/4,235)	*mcr-3* (1)<br>[Ec, *mcr-1* & *mcr-3*]<br>*mcr-4 - mcr-9* (0)<br>*mcr-10* n.t.	[88]
THA	2016-2017	• **P – healthy, longitudinal study (4 farms)**	Ec: 100 ColR	64% (64/100)<br>[24 Ec *mcr-1* & *mcr-2*]	38% (38/100)<br>[24 Ec *mcr-1* & *mcr-2*]	n.t.	[89]

Country[a]	Year of isolation	Source (no.) of samples[b]	Bacterial species[c], no. of isolates (no. of ColR isolates)[d]	*mcr-1* % (no. of positive isolates per no. of tested isolates)	*mcr-2*	*mcr-3 – mcr-10* no. of pos. isolates[e]	REF
THA	2017-2020	• **P – healthy, longitudinal study (1 farm), fecal samples (70)**<br>• Wastewater (50)<br>• H – farm workers, rectal swabs (50)	Ec: 33 (ColR)	P: 24.3% (17/70)<br>[2 *mcr-1* & *mcr-3*]<br>Wastewater: 18% (9/50)<br>H: 8% (4/50)	0% (0/170)	*mcr-3* (5)<br>[2 *mcr-1* & *mcr-3*]<br>*mcr-4, -5, -6, -7, -8* (0)<br>*mcr-9 – mcr-10* n.t.	[90]
THA	2018	• H – healthy farmer, stool	Kp: 1 ColR (case report)	0% (0/1)	100% (1/1)<br>[*mcr-1* & *mcr-3*]	*mcr-3* (1)<br>[*mcr-1* & *mcr-3*]<br>*mcr-4 – mcr-10* n.t.	[91]
THA	not specified	• H – healthy, feces (3)	Ec: 3 ColR	66.7% (2/3)<br>[1 *mcr-1* & *mcr-3*]	33.3% (1/3)<br>[*mcr-2* & *mcr-3*]	*mcr-3* (2)<br>(1 *mcr-1* & *mcr-3*, 1 *mcr-2* & *mcr-3*]<br>*mcr-4 – mcr-10* n.t.	[92]
TUN	2011-2013	• Camel calves – healthy (23), diarrhea (29); 25 extensive camel farms	Ec: 51 ColS<br>Ecl: 1 ColR	0% (0/52)	0% (0/52)	n.t.	[93]
TUR	not specified	• CA, sheep	Ec: 49 O157	0% (0/49)	6.1% (3/49)<br>[3 *mcr-2* & *mcr-3*]	*mcr-3* (5)<br>[3 *mcr-2* & *mcr-3*]<br>*mcr-4, -5* (0)<br>*mcr-6 – mcr-10* n.t.	[94]
TUR	not specified	• H - clinical	Kp: 38	0% (0/38)	0% (0/38)	*mcr-3* (0)<br>*mcr-4 – mcr-10* n.t.	[95]
TUR	2015-2016	• H – clinical	Ent: 329 (Kp: 217, Sal 75, Ec 31, Ecl 3, Kox 2, Eaer 1)	0% (0/329)	0% (0/329)	n.t.	[96]
TUR	2018-2021	• H – clinical	Kp: 150 CPE (78 ColR)	0% (0/150)	0% (0/150)	*mcr-3 – mcr-5* (0)<br>*mcr-6 – mcr-10* n.t.	[97]
TUR	2021	• H – inpatients in ICU, oral swabs (96)	Ab: 21 (0 ColR)	0% (0/21)	4.8% (1/21)	*mcr-3 – mcr-5* (0)<br>*mcr-6 – mcr-10* n.t.	[98]
USA	2006-2014	• **Livestock (180),** wildlife (320), watersheds (240), leafy vegetables (220), sediment, soil, fruit, other vegetables (10 each)	Ec: 1,000 STEC	0% (0/1,000)	0% (0/1,000)	n.t.	[99]
USA	not specified	• H (109)<br>• nonhuman isolates (2)	Ent: 111 (Kp: 61, Ec: 33, Ecl: 7, Kox: 2, Ka: 2, Cb: 2, Ea:1, Sal: 3) (28 ColR)	13.5% (15/111)<br>[Ec: 11, Kp: 1, Sal: 3]	0.9% (1/111)<br>[Ec: 1]	*mcr-3 – mcr-10* n.t.	[100]
VNM	2011	• CH – healthy, feces (51)	Ec potential ESBL/AmpC: 126 (11 ColR)	VNM: 100% (11/11 ColR)	0% (0/11)	n.t.	[51]
VNM	2015-2017	• Food samples – CH (116), **pork (112)**, fish (112), shrimp (112)	CH – Ec ESBL: 77<br>Pork – Ec ESBL: 62<br>Fish – Ec ESBL: 47	CH: 53.2% (41/77)<br>Pork: 11.3% (7/62)<br>Fish: 6.4% (3/47)	0% (0/208)	*mcr-3* (9)<br>[8 *mcr-1* & *mcr-3*]<br>*mcr-4 – mcr-8* (0)	[101]

Country[a]	Year of isolation	Source (no.) of samples[b]	Bacterial species[c], no. of isolates (no. of ColR isolates) [d]	*mcr-1*	*mcr-2*	*mcr-3 – mcr-10*	REF
				% (no. of positive isolates per no. of tested isolates)		no. of pos. isolates[e]	
			Shrimp – Ec ESBL: 22	Shrimp: 22.7% (5/22) [8 *mcr-1* & *mcr-3*]		*mcr-9, -10* n.t.	
ZAF	2016	• POUL - clinical	Ec: 50 (8 isolates with maximum colistin MIC (2 µg/mL) in this study)	12.5% (1/8)	0% (0/8)	n.t.	[102]
ZAF	2017-2018	• POUL – healthy broilers, fecal samples (2400)	Cj: 26	0% (0/26)	0% (0/26)	*mcr-3, -5* (0)<br>*mcr-4* (8)<br>*mcr-6 – mcr-10* n.t.	[103]
WW	2014-2016	• H – clinical (INFORM global surveillance program (44,407 isolates from 39 countries)	Ent: 908 ColR	2.6% (24/908)	0% (0/908)	*mcr-3* (2)<br>*mcr-4* (0)<br>*mcr-5* (1)<br>*mcr-6 – mcr-10* n.t.	[104]

[a] 3-letter country abbreviation: BEL, Belgium; BGD, Bangladesh; BOL, Bolivia; BRA, Brazil; Can, Canada; CHE, Switzerland; CHN, China; CZE, Czechia; DEU, Germany; DNK, Denmark; EGY, Egypt; ESP, Spain; EUR, Europe; FRA, France; GBR, United Kingdom; GHA, Ghana; HKG, Hong Kong; IND, India; IRN, Iran; IRQ, Iraq; ITA, Italy; JPN, Japan; KHM, Cambodia; KOR, The Republic of Korea; MYS, Malaysia; NLD, Netherlands; PAK, Pakistan; POL, Poland; ROU, Romania; SEN, Senegal; SGP, Singapore; THA, Thailand; TUN, Tunisia; TUR, Turkey; USA, United States of America; VNM, Viet Nam; ZAF, South Africa. WW, worldwide.

[b] Abbreviations of hosts: CA, cattle; CH, chicken; H, human; MB, migratory birds; P, pig; POUL, poultry; PWD, post weaning diarrhea; RB, resident wild bird; SW, surface water; UTI, urinary tract infection. Samples from pig or pig meat are marked in bold.

[c] Abbreviations of bacterial species and pathotypes: Ab, *Acinetobacter baumannii*; Am, *Aeromonas hydrophila*; Bcep, *Burkholderia cepacia*; Cb, *Citrobacter* species; Cj, *Campylobacter jejuni*; Ea, *Escherichia albertii*; Ec, *Escherichia coli*; iEc, indicator *E. coli*, Ecl, *Enterobacter cloacae*; Ent, Enterobacterales; GN, Gram negative bacteria; Hal, *Hafnia alvei*; Ka, *Klebsiella aerogenes*; Kox, *Klebsiella oxytoca*; Kp, *Klebsiella pneumoniae*; Pa, *Pseudomonas aeruginosa*; Pm, *Proteus mirabilis*; Rorn, *Raoultella ornithinolytica*, Sal, *Salmonella enterica*; APEC, Avian Pathogenic *E. coli*; MPEC, Mammary Pathogenic *E. coli*; STEC, Shiga toxin producing *E. coli*;

[d] If available, the number of ColR isolates is provided. Unless otherwise stated, ColR means acquired Col-resistance, excluding *Serratia* spp., *Providencia* spp., *Proteus* spp., and *Morganella* spp., which are intrinsically colistin non-susceptible). Abbreviations: 3GCR, 3rd-generation cephalosporin resistant bacteria; AmpC, AmpC β-Lactamase; APEC, Avian Pathogenic *E. coli*; Col-non-S, Colistin non-susceptible; ColR, colistin resistant; CPE, carbapenem-resistant Enterobacteriaceae; CPOs, carbapenemase-producing organism; CPR, carbapenem-resistant; ESBL, Extended-spectrum β-Lactamase; ETEC, enterotoxigenic *E. coli*; STEC, Shiga toxin producing *E. coli*;

[e] Unless otherwise stated, *mcr-6* to *mcr-10* genes were not investigated in the studies cited, e.g., due the fact, that these *mcr* gene variants were unknown at the time the studies were performed.

frontiers

References

1. Xavier, B.B., et al., *Identification of a novel plasmid-mediated colistin-resistance gene, mcr-2, in Escherichia coli, Belgium, June 2016.* Euro Surveill, 2016. **21**(27).
2. Garcia-Graells, C., et al., *Detection of Plasmid-Mediated Colistin Resistance, mcr-1 and mcr-2 genes, in Salmonella spp. Isolated from Food at Retail in Belgium from 2012 to 2015.* Foodborne Pathog Dis, 2018. **15**(2): p. 114-117.
3. Timmermans, M., et al., *Colistin resistance genes mcr-1 to mcr-5, including a case of triple occurrence (mcr-1, -3 and -5), in Escherichia coli isolates from faeces of healthy pigs, cattle and poultry in Belgium, 2012-2016.* Int J Antimicrob Agents, 2021. **57**(6): p. 106350.
4. Torres, D.A., et al., *Colistin resistance in Gram-negative bacteria analysed by five phenotypic assays and inference of the underlying genomic mechanisms.* BMC Microbiol, 2021. **21**(1): p. 321.
5. Buess, S., et al., *Assessment of animals as a reservoir for colistin resistance: No MCR-1/MCR-2-producing Enterobacteriaceae detected in Swiss livestock.* J Glob Antimicrob Resist, 2017. **8**: p. 33-34.
6. Liassine, N., et al., *Very low prevalence of MCR-1/MCR-2 plasmid-mediated colistin resistance in urinary tract Enterobacteriaceae in Switzerland.* Int J Infect Dis, 2016. **51**: p. 4-5.
7. Zurfluh, K., et al., *Screening for fecal carriage of MCR-producing Enterobacteriaceae in healthy humans and primary care patients.* Antimicrob Resist Infect Control, 2017. **6**: p. 28.
8. Tkadlec, J., et al., *The Intestinal Carriage of Plasmid-Mediated Colistin-Resistant Enterobacteriaceae in Tertiary Care Settings.* Antibiotics (Basel), 2021. **10**(3).
9. Roschanski, N., et al., *Retrospective survey of mcr-1 and mcr-2 in German pig-fattening farms, 2011-2012.* Int J Antimicrob Agents, 2017. **50**(2): p. 266-271.
10. Borowiak, M., et al., *Development of a Novel mcr-6 to mcr-9 Multiplex PCR and Assessment of mcr-1 to mcr-9 Occurrence in Colistin-Resistant Salmonella enterica Isolates From Environment, Feed, Animals and Food (2011-2018) in Germany.* Front Microbiol, 2020. **11**: p. 80.
11. Kneis, D., T.U. Berendonk, and S. Hess, *High prevalence of colistin resistance genes in German municipal wastewater.* Sci Total Environ, 2019. **694**: p. 133454.
12. Litrup, E., et al., *Plasmid-borne colistin resistance gene mcr-3 in Salmonella isolates from human infections, Denmark, 2009-17.* Euro Surveill, 2017. **22**(31).
13. Roer, L., et al., *Novel mcr-3 variant, encoding mobile colistin resistance, in an ST131 Escherichia coli isolate from bloodstream infection, Denmark, 2014.* Euro Surveill, 2017. **22**(31).
14. Migura-Garcia, L., et al., *mcr-Colistin Resistance Genes Mobilized by IncX4, IncHI2, and IncI2 Plasmids in Escherichia coli of Pigs and White Stork in Spain.* Front Microbiol, 2019. **10**: p. 3072.
15. Garcia-Menino, I., et al., *Genomic Characterization of Prevalent mcr-1, mcr-4, and mcr-5 Escherichia coli Within Swine Enteric Colibacillosis in Spain.* Front Microbiol, 2019. **10**: p. 2469.
16. Garcia, V., et al., *Co-occurrence of mcr-1, mcr-4 and mcr-5 genes in multidrug-resistant ST10 Enterotoxigenic and Shiga toxin-producing Escherichia coli in Spain (2006-2017).* Int J Antimicrob Agents, 2018. **52**(1): p. 104-108.
17. Vela, A.I., et al., *Moraxella pluranimalium sp. nov., isolated from animal specimens.* Int J Syst Evol Microbiol, 2009. **59**(Pt 4): p. 671-4.
18. Poirel, L., et al., *MCR-2-mediated plasmid-borne polymyxin resistance most likely originates from Moraxella pluranimalium.* J Antimicrob Chemother, 2017. **72**(10): p. 2947-2949.
19. Hernandez, M., et al., *Co-occurrence of colistin-resistance genes mcr-1 and mcr-3 among multidrug-resistant Escherichia coli isolated from cattle, Spain, September 2015.* Euro Surveill, 2017. **22**(31).
20. Miguela-Villoldo, P., et al., *Complementarity of Selective Culture and qPCR for Colistin Resistance Screening in Fresh and Frozen Pig Cecum Samples.* Front Microbiol, 2020. **11**: p. 572712.

21. El Garch, F., et al., *mcr-1-like detection in commensal Escherichia coli and Salmonella spp. from food-producing animals at slaughter in Europe.* Vet Microbiol, 2018. **213**: p. 42-46.
22. Saly, M., et al., *Prevalence of faecal carriage of colistin-resistant Gram-negative rods in a university hospital in western France, 2016.* J Med Microbiol, 2017. **66**(6): p. 842-843.
23. Um, M.M., et al., *High Fecal Prevalence of mcr-Positive Escherichia coli in Veal Calves at Slaughter in France.* Antibiotics (Basel), 2022. **11**(8).
24. Hamame, A., et al., *Screening of Colistin-Resistant Bacteria in Domestic Pets from France.* Animals (Basel), 2022. **12**(5).
25. Hamame, A., et al., *Genomic characterisation of an mcr-1 and mcr-3-producing Escherichia coli strain isolated from pigs in France.* J Glob Antimicrob Resist, 2022. **28**: p. 174-179.
26. AbuOun, M., et al., *mcr-1 and mcr-2 variant genes identified in Moraxella species isolated from pigs in Great Britain from 2014 to 2015.* J Antimicrob Chemother, 2017. **72**(10): p. 2745-2749.
27. Sia, C.M., et al., *The characterization of mobile colistin resistance (mcr) genes among 33000 Salmonella enterica genomes from routine public health surveillance in England.* Microb Genom, 2020. **6**(2).
28. Dobrzanska, D.A., et al., *Preventive antibiotic treatment of calves: emergence of dysbiosis causing propagation of obese state-associated and mobile multidrug resistance-carrying bacteria.* Microb Biotechnol, 2020. **13**(3): p. 669-682.
29. Bertelloni, F., et al., *Low Level of Colistin Resistance and mcr Genes Presence in Salmonella spp.: Evaluation of Isolates Collected between 2000 and 2020 from Animals and Environment.* Antibiotics (Basel), 2022. **11**(2).
30. Venditti, C., et al., *Letter to the Editor: Surveillance of mcr-1 and mcr-2 genes in Carbapenem-resistant Klebsiella pneumoniae strains from an Italian Hospital.* Euro Surveill, 2017. **22**(35).
31. Alba, P., et al., *Molecular Epidemiology of mcr-Encoded Colistin Resistance in Enterobacteriaceae From Food-Producing Animals in Italy Revealed Through the EU Harmonized Antimicrobial Resistance Monitoring.* Front Microbiol, 2018. **9**: p. 1217.
32. Curcio, L., et al., *Detection of the colistin resistance gene mcr-1 in pathogenic Escherichia coli from pigs affected by post-weaning diarrhoea in Italy.* J Glob Antimicrob Resist, 2017. **10**: p. 80-83.
33. Carattoli, A., et al., *Novel plasmid-mediated colistin resistance mcr-4 gene in Salmonella and Escherichia coli, Italy 2013, Spain and Belgium, 2015 to 2016.* Euro Surveill, 2017. **22**(31).
34. Del Bianco, F., et al., *Microbiological surveillance of plasmid mediated colistin resistance in human Enterobacteriaceae isolates in Romagna (Northern Italy): August 2016-July 2017.* Int J Infect Dis, 2018. **69**: p. 96-98.
35. Casagrande Proietti, P., et al., *mcr-1-Mediated Colistin Resistance and Genomic Characterization of Antimicrobial Resistance in ESBL-Producing Salmonella Infantis Strains from a Broiler Meat Production Chain in Italy.* Antibiotics (Basel), 2022. **11**(6).
36. Cilia, G., et al., *Phenotypic and genotypic resistance to colistin in E. coli isolated from wild boar (Sus scrofa) hunted in Italy.* European Journal of Wildlife Research, 2021. **67**(57).
37. Terveer, E.M., et al., *Prevalence of colistin resistance gene (mcr-1) containing Enterobacteriaceae in feces of patients attending a tertiary care hospital and detection of a mcr-1 containing, colistin susceptible E. coli.* PLoS One, 2017. **12**(6): p. e0178598.
38. Schrauwen, E.J.A., et al., *High prevalence of the mcr-1 gene in retail chicken meat in the Netherlands in 2015.* Antimicrob Resist Infect Control, 2017. **6**: p. 83.
39. Zajac, M., et al., *Occurrence and Characterization of mcr-1-Positive Escherichia coli Isolated From Food-Producing Animals in Poland, 2011-2016.* Front Microbiol, 2019. **10**: p. 1753.
40. Maciuca, I.E., et al., *Genetic Features of mcr-1 Mediated Colistin Resistance in CMY-2-Producing Escherichia coli From Romanian Poultry.* Front Microbiol, 2019. **10**: p. 2267.
41. Dutta, A., et al., *Acquisition of Plasmid-Mediated Colistin Resistance Gene mcr-1 in Escherichia coli of Livestock Origin in Bangladesh.* Microb Drug Resist, 2020. **26**(9): p. 1058-1062.

42. Dutta, A., et al., *An investigation of plasmid-mediated colistin resistance mechanism, MCR in Escherichia coli of human, veterinary and environmental origin in Bangladesh.* International Journal of Infectious Diseases, 2018. **73, Supplement**.
43. Ara, B., et al., *Detection of mobile colistin-resistance gene variants (mcr-1 and mcr-2) in urinary tract pathogens in Bangladesh: the last resort of infectious disease management colistin efficacy is under threat.* Expert Rev Clin Pharmacol, 2021. **14**(4): p. 513-522.
44. Amin, M.B., et al., *Occurrence and genetic characteristics of mcr-1-positive colistin-resistant E. coli from poultry environments in Bangladesh.* J Glob Antimicrob Resist, 2020. **22**: p. 546-552.
45. Islam, S., et al., *High abundance of the colistin resistance gene mcr-1 in chicken gut-bacteria in Bangladesh.* Sci Rep, 2020. **10**(1): p. 17292.
46. Ahmed, S., et al., *High prevalence of mcr-1-encoded colistin resistance in commensal Escherichia coli from broiler chicken in Bangladesh.* Sci Rep, 2020. **10**(1): p. 18637.
47. Giani, T., et al., *High prevalence of carriage of mcr-1-positive enteric bacteria among healthy children from rural communities in the Chaco region, Bolivia, September to October 2016.* Euro Surveill, 2018. **23**(45).
48. Moreno, L.Z., et al., *First report of mcr-1-harboring Salmonella enterica serovar Schwarzengrund isolated from poultry meat in Brazil.* Diagn Microbiol Infect Dis, 2019. **93**(4): p. 376-379.
49. Barbieri, N.L., et al., *mcr-1 Identified in Fecal Escherichia coli and Avian Pathogenic E. coli (APEC) From Brazil.* Front Microbiol, 2021. **12**: p. 659613.
50. Hallenberg, G.S., et al., *Detection of mcr-Mediated Colistin Resistance in Escherichia coli Isolates from Pigs in Small-Scale Farms in Cambodia.* Antimicrobial Agents and Chemotherapy, 2019. **63**(3).
51. Vounba, P., et al., *Prevalence of colistin resistance and mcr-1/mcr-2 genes in extended-spectrum beta-lactamase/AmpC-producing Escherichia coli isolated from chickens in Canada, Senegal and Vietnam.* J Glob Antimicrob Resist, 2019. **19**: p. 222-227.
52. Rhouma, M., et al., *First identification of mcr-1/mcr-2 genes in the fecal microbiota of Canadian commercial pigs during the growing and finishing period.* Vet Med (Auckl), 2019. **10**: p. 65-67.
53. Yassin, A.K., et al., *Identification and characterization of mcr mediated colistin resistance in extraintestinal Escherichia coli from poultry and livestock in China.* FEMS Microbiol Lett, 2017. **364**(24).
54. Wu, C., et al., *Rapid rise of the ESBL and mcr-1 genes in Escherichia coli of chicken origin in China, 2008-2014.* Emerg Microbes Infect, 2018. **7**(1): p. 30.
55. Wang, X., et al., *Molecular epidemiology of colistin-resistant Enterobacteriaceae in inpatient and avian isolates from China: high prevalence of mcr-negative Klebsiella pneumoniae.* Int J Antimicrob Agents, 2017. **50**(4): p. 536-541.
56. Zhang, J., et al., *Housefly (Musca domestica) and Blow Fly (Protophormia terraenovae) as Vectors of Bacteria Carrying Colistin Resistance Genes.* Appl Environ Microbiol, 2018. **84**(1).
57. Zhang, J., et al., *Molecular detection of colistin resistance genes (mcr-1, mcr-2 and mcr-3) in nasal/oropharyngeal and anal/cloacal swabs from pigs and poultry.* Sci Rep, 2018. **8**(1): p. 3705.
58. Zhang, X., et al., *Colistin resistance prevalence in Escherichia coli from domestic animals in intensive breeding farms of Jiangsu Province.* Int J Food Microbiol, 2019. **291**: p. 87-90.
59. Zhang, J., et al., *Molecular detection of colistin resistance genes (mcr-1 to mcr-5) in human vaginal swabs.* BMC Res Notes, 2018. **11**(1): p. 143.
60. Zheng, B., et al., *Occurrence and Genomic Characterization of ESBL-Producing, MCR-1-Harboring Escherichia coli in Farming Soil.* Front Microbiol, 2017. **8**: p. 2510.
61. Wang, G., et al., *Colistin-resistance mcr genes in Klebsiella pneumoniae from companion animals.* J Glob Antimicrob Resist, 2021. **25**: p. 35-36.
62. Ahmed, Z.S., et al., *Evidence of colistin resistance genes (mcr-1 and mcr-2) in wild birds and its public health implication in Egypt.* Antimicrob Resist Infect Control, 2019. **8**: p. 197.

63. Tartor, Y.H., et al., *Virulence Determinants and Plasmid-Mediated Colistin Resistance mcr Genes in Gram-Negative Bacteria Isolated From Bovine Milk.* Front Cell Infect Microbiol, 2021. **11**: p. 761417.
64. Tartor, Y.H., et al., *Whole-Genome Sequencing of Gram-Negative Bacteria Isolated From Bovine Mastitis and Raw Milk: The First Emergence of Colistin mcr-10 and Fosfomycin fosA5 Resistance Genes in Klebsiella pneumoniae in Middle East.* Front Microbiol, 2021. **12**: p. 770813.
65. Rabie, R.A. and A.L. Abdallah, *Plasmid mediated colistin resistant genes mcr-1 and mcr-2 among Escherichia coli and Klebsiella pneumoniae isolates at Zagazig University Hospitals, Egypt* Egyptian Journal of Medical Microbiology 2020. **29**(1): p. 61-66.
66. Meheissen, M.A., et al., *Colistin resistance and heteroresistance in Klebsiella pneumoniae & Escherichia coli clinical isolates from intensive care units.* Epidemiol Mikrobiol Imunol, 2022. **71**(2): p. 86-92.
67. Deku, J.G., et al., *Carbapenemase production and detection of Colistin-resistant genes in clinical isolates of Escherichia coli from the Ho Teaching Hospital, Ghana.* Can J Infect Dis Med Microbiol, 2022. **2022**: p. 1544624.
68. Chan, W.S., et al., *Prospective study on human fecal carriage of Enterobacteriaceae possessing mcr-1 and mcr-2 genes in a regional hospital in Hong Kong.* BMC Infect Dis, 2018. **18**(1): p. 81.
69. Pragasam, A.K., et al., *Molecular Mechanisms of Colistin Resistance in Klebsiella pneumoniae Causing Bacteremia from India-A First Report.* Front Microbiol, 2016. **7**: p. 2135.
70. Mitra, S., et al., *Colistin resistance in Gram-negative ocular infections: prevalence, clinical outcome and antibiotic susceptibility patterns.* Int Ophthalmol, 2020. **40**(5): p. 1307-1317.
71. Kar, P., et al., *Detection of Colistin Resistance in Carbapenem Resistant Enterobacteriaceae by Reference Broth Microdilution and Comparative Evaluation of Three Other Methods.* J Lab Physicians, 2021. **13**(3): p. 263-269.
72. Ilbeigi, K., et al., *Molecular survey of mcr1 and mcr2 plasmid mediated colistin resistance genes in Escherichia coli isolates of animal origin in Iran.* BMC Res Notes, 2021. **14**(1): p. 107.
73. Moosavian, M. and N. Emam, *The first report of emerging mobilized colistin-resistance (mcr) genes and ERIC-PCR typing in Escherichia coli and Klebsiella pneumoniae clinical isolates in southwest Iran.* Infect Drug Resist, 2019. **12**: p. 1001-1010.
74. Nikkhahi, F., et al., *First detection of mobilized colistin resistance mcr-1 gene in Escherichia coli isolated from livestock and sewage in Iran.* New Microbes New Infect, 2021. **41**: p. 100862.
75. Askari Badouei, M., et al., *High prevalence of clonally related multiple resistant Salmonella Infantis carrying class 1 integrons in broiler farms.* Vet Ital, 2021. **57**(3).
76. Al-Kadmy, I.M.S., et al., *Prevalence of Genes Involved in Colistin Resistance in Acinetobacter baumannii: First Report from Iraq.* Microb Drug Resist, 2020. **26**(6): p. 616-622.
77. Do, K.H., et al., *Virulence and antimicrobial resistance profiles of Escherichia coli encoding mcr gene from diarrhoeic weaned piglets in Korea during 2007-2016.* Journal of Global Antimicrobial Resistance, 2020. **20**: p. 324-327.
78. Kawanishi, M., et al., *Prevalence of Colistin Resistance Gene mcr-1 and Absence of mcr-2 in Escherichia coli Isolated from Healthy Food-Producing Animals in Japan.* Antimicrob Agents Chemother, 2017. **61**(1).
79. Fukuda, A., et al., *High prevalence of mcr-1, mcr-3 and mcr-5 in Escherichia coli derived from diseased pigs in Japan.* Int J Antimicrob Agents, 2018. **51**(1): p. 163-164.
80. Nishino, Y., et al., *Detection of the mcr-1 gene in colistin-resistant Escherichia coli from retail meat in Japan.* Microbiol Immunol, 2017. **61**(12): p. 554-557.
81. Aklilu, E. and K. Raman, *MCR-1 Gene Encoded Colistin-Resistant Escherichia coli in Raw Chicken Meat and Bean Sprouts in Malaysia.* Int J Microbiol, 2020. **2020**: p. 8853582.
82. Javed, H., et al., *Emergence of plasmid-mediated mcr genes from Gram-negative bacteria at the human-animal interface.* Gut Pathog, 2020. **12**(1): p. 54.

83. Ejaz, H., et al., *Molecular Epidemiology of Extensively Drug-Resistant mcr Encoded Colistin-Resistant Bacterial Strains Co-Expressing Multifarious beta-Lactamases.* Antibiotics (Basel), 2021. **10**(4).
84. Imtiaz, W., et al., *Analysis of Antibiotic Resistance and Virulence Traits (Genetic and Phenotypic) in Klebsiella pneumoniae Clinical Isolates from Pakistan: Identification of Significant Levels of Carbapenem and Colistin Resistance.* Infect Drug Resist, 2021. **14**: p. 227-236.
85. La, M.V., et al., *Prevalence and antibiotic susceptibility of colistin-resistance gene (mcr-1) positive Enterobacteriaceae in stool specimens of patients attending a tertiary care hospital in Singapore.* Int J Infect Dis, 2019. **85**: p. 124-126.
86. Trongjit, S. and R. Chuanchuen, *Whole genome sequencing and characteristics of Escherichia coli with co-existence of ESBL and mcr genes from pigs.* PLoS One, 2021. **16**(11): p. e0260011.
87. Trongjit, S., et al., *Plasmid-mediated colistin resistance and ESBL production in Escherichia coli from clinically healthy and sick pigs.* Sci Rep, 2022. **12**(1): p. 2466.
88. Paveenkittiporn, W., et al., *Whole-Genome Sequencing of Clinically Isolated Carbapenem-Resistant Enterobacterales Harboring mcr Genes in Thailand, 2016-2019.* Front Microbiol, 2021. **11**: p. 586368.
89. Ketkhao, P., et al., *Antimicrobial resistance profiles of Escherichia coli from swine farms using different antimicrobials and management systems.* Vet World, 2021. **14**(3): p. 689-695.
90. Khine, N.O., et al., *Longitudinal Monitoring Reveals Persistence of Colistin-Resistant Escherichia coli on a Pig Farm Following Cessation of Colistin Use.* Front Vet Sci, 2022. **9**: p. 845746.
91. Stosic, M.S., et al., *Novel mcr-3.40 variant co-located with mcr-2.3 and* $bla_{CTX\text{-}M\text{-}63}$ *on an IncHI1B/IncFIB plasmid found in Klebsiella pneumoniae from a healthy carrier in Thailand.* J Antimicrob Chemother, 2021.
92. Phuadraksa, T., et al., *Co-occurrence of mcr-2 and mcr-3 genes on chromosome of multidrug-resistant Escherichia coli isolated from healthy individuals in Thailand.* Int J Antimicrob Agents, 2022: p. 106662.
93. Rhouma, M., et al., *Screening for fecal presence of colistin-resistant Escherichia coli and mcr-1 and mcr-2 genes in camel-calves in southern Tunisia.* Acta Vet Scand, 2018. **60**(1): p. 35.
94. Ayaz, N.D., et al., *Plasmid-Mediated Colistin Resistance in Escherichia coli O157:H7 Cattle and Sheep Isolates and Whole-Genome Sequence of a Colistin-Resistant Sorbitol Fermentative Escherichia coli O157:H7.* Microb Drug Resist, 2019. **25**(10): p. 1497-1506.
95. Hosbul, T., et al., *Carbapenem and Colistin Resistant Klebsiella Pneumoniae ST14 and ST2096 Dominated in Two Hospitals in Turkey.* Clin Lab, 2021. **67**(9).
96. Sari, A.N., et al., *[Results of a multicenter study investigating plasmid mediated colistin resistance genes (mcr-1 and mcr-2) in clinical Enterobacteriaceae isolates from Turkey].* Mikrobiyol Bul, 2017. **51**(3): p. 299-303.
97. Hosbul, T., et al., *[In Vitro Activity of Ceftazidime-avibactam and Colistin Against Carbapenem-Resistant Klebsiella pneumoniae Clinical Isolates].* Mikrobiyol Bul, 2022. **56**(2): p. 218-229.
98. Duman, Y., et al., *Oral colonization of Acinetobacter baumannii in intensive care units: Risk factors, incidence, molecular epidemiology, association with the occur of pneumonia and sepsis, and infection control measures.* Iran J Basic Med Sci, 2022. **25**(2): p. 239-244.
99. Mavrici, D., et al., *Screening for the presence of mcr-1/mcr-2 genes in Shiga toxin-producing Escherichia coli recovered from a major produce-production region in California.* PLoS One, 2017. **12**(11): p. e0187827.
100. Lutgring, J.D., et al., *Evaluation of the MicroScan Colistin Well and Gradient Diffusion Strips for Colistin Susceptibility Testing in Enterobacteriaceae.* J Clin Microbiol, 2019. **57**(5).
101. Le, P.Q., et al., *Prevalence of mobile colistin resistance (mcr) genes in extended-spectrum beta-lactamase-producing Escherichia coli isolated from retail raw foods in Nha Trang, Vietnam.* Int J Food Microbiol, 2021. **346**: p. 109164.

102. Hassan, I.Z., et al., *Antimicrobial resistance and mcr-1 gene in Escherichia coli isolated from poultry samples submitted to a bacteriology laboratory in South Africa.* Vet World, 2021. **14**(10): p. 2662-2669.
103. Ramatla, T., et al., *Campylobacter jejuni from Slaughter Age Broiler Chickens: Genetic Characterization, Virulence, and Antimicrobial Resistance Genes.* Int J Microbiol, 2022. **2022**: p. 1713213.
104. Wise, M.G., et al., *Prevalence of mcr-type genes among colistin-resistant Enterobacteriaceae collected in 2014-2016 as part of the INFORM global surveillance program.* PLoS One, 2018. **13**(4): p. e0195281.
